# Nutritional Composition and Anti-Type 2 Diabetes Mellitus Potential of Femur Bone Extracts from Bovine, Chicken, Sheep, and Goat: Phytochemical and In Vivo Studies

**DOI:** 10.3390/nu15184037

**Published:** 2023-09-18

**Authors:** Naseh A. Algehainy, Esraa M. Mohamed, Hanan F. Aly, Eman A. Younis, Faisal H. Altemani, Mohammad A. Alanazi, Gerhard Bringmann, Usama Ramadan Abdelmohsen, Abeer H. Elmaidomy

**Affiliations:** 1 Department of Medical Laboratory Technology, Faculty of Applied Medical Sciences, University of Tabuk, Tabuk 71491, Saudi Arabia; nalgehainy@ut.edu.sa (N.A.A.); faltemani@ut.edu.sa (F.H.A.); m.alenezi@ut.edu.sa (M.A.A.); 2 Department of Pharmacognosy, Faculty of Pharmacy, Misr University for Science & Technology (MUST), Giza 12566, Egypt; esraakadrymohamed@gmail.com; 3 Department of Therapeutic Chemistry, National Research Centre (NRC), El-Bouth St., Cairo 12622, Egypt; hanan_abduallah@yahoo.com (H.F.A.); youniseman530@yahoo.com (E.A.Y.); 4 Institute of Organic Chemistry, University of Würzburg, Am Hubland, 97074 Würzburg, Germany; 5 Department of Pharmacognosy, Faculty of Pharmacy, Deraya University, 7 Universities Zone, New Minia 61111, Egypt; 6 Department of Pharmacognosy, Faculty of Pharmacy, Beni-Suef University, Beni-Suef 62511, Egypt; abeer011150@pharm.bsu.edu.eg

**Keywords:** nutritional deficiencies, TNF-α, sICAM-1, sVCAM-1

## Abstract

Nutritional deficits in one’s diet have been established as the key risk factor for T2DM in recent years. Nutritional therapy has been demonstrated to be useful in treating T2DM. The current study was carried out to assess the nutritional composition of bovine (12 months), chicken (4 months), sheep (13 months), and goat (9 months) femur bone extracts, as well as their potential therapeutic effects on T2DM regression in a Wistar albino rat model (500 mg/kg b.wt.). The proximate composition of the different extracts, their fatty acid composition, their amino acids, and their mineral contents were identified. In vivo data indicated considerably improved T2DM rats, as seen by lower serum levels of TL, TG, TC, ALT, AST, ALP, bilirubin, creatinine, urea, IL-6, TNF-α, sICAM-1, sVCAM-1, and MDA. Low levels of HDL-C, GSH, and total proteins were restored during this study. Histological investigations of liver and pancreatic tissue revealed that the distribution of collagen fibers was nearly normal. The bovine extract, on the other hand, was the most active, followed by the sheep, goat, and finally chicken extract. This research could result in the creation of a simple, noninvasive, low-cost, and reliable method for T2DM control, paving the way for potential early therapeutic applications in T2DM control.

## 1. Introduction

Regarding the bones of animals, their chemical composition plays a significant role in sustaining optimum health by providing necessary nutrients [1]. Animal bones contain collagen, amino acids, and minerals. Being a fibrous protein, collagen is an essential component of maintaining the structures of different organs and tissues [2]. The physiological functions of the human body are significantly influenced by the amino acid content, which influences the preservation of good health, directly or indirectly. The mineral composition of bone consists mostly of calcium (Ca) and phosphorus (P). Calcium protects against osteoporosis by promoting bone growth and decreasing bone loss [3]. Furthermore, bone marrow may have a positive effect on the immune system since it aids in the transfer of oxygen to the cells of the body. The marrow includes critical minerals such as P, Ca, and Fe (iron), which are required for bone maintenance [4].

Bone broth has long been known to have health advantages, but it was not until a decade ago that its curative power was scientifically proven [5]. For instance, it has been discovered that the widely believed ability of chicken soup to treat symptomatic upper respiratory tract infections results from either an increase in the velocity of nasal mucus or its modest anti-inflammatory properties [6]. For patients with gut and psychology syndrome (GAPS), including those with autism and attention-deficit hyperactivity disorder (ADHD), bone broth has been endorsed more frequently in recent years [7]. Some people view bone broths as a crucial nutritional source of nutrients, like calcium, which is especially favored by people who are lactose intolerant or cannot consume milk products. For instance, in some Asian cultures, bone broths made from soaking chicken or other bones in vinegar have historically been recommended for calcium or iron enrichment, especially during pregnancy and the time after childbirth [8]. Dietitians are also widely promoting bone broth as a calcium supplement.

One of the most prevalent metabolic diseases in the world, Type 2 diabetes mellitus (T2DM), is predominantly caused by the coincidence of two key factors: the faulty pancreatic beta-cell (β-cell) production of insulin and insulin resistance in insulin-sensitive tissues [9]. The molecular mechanisms involved in insulin synthesis and release, as well as the insulin response in tissues, must be strictly managed since insulin release and action must perfectly match the metabolic demand [10]. A metabolic imbalance can thus be caused by flaws in any of the relevant processes, which then results in the pathophysiology of T2DM [9]. Epidemiological data show concerning trends that point to a bleak future for T2DM. According to the International Diabetes Federation (IDF), diabetes killed 4.2 million people in 2019 and affected 463 million adults aged 20 to 79; this figure is expected to rise to 700 million in 2045 [10]. In 2019, at least USD 720 billion in health care expenses were directly related to diabetes [10]. It is even possible that the true illness burden associated with T2DM is underestimated, as one in every three diabetics, or 232 million people, is yet undiagnosed. Diabetes affects the greatest number of people between the ages of 40 and 59. T2DM incidence and prevalence vary geographically, with more than 80% of patients living in low- to middle-income countries, posing additional challenges to effective treatment [10]. Cardiovascular disease (CVD) is the main cause of morbidity and mortality among T2DM patients, with a 15% increased risk of all-cause mortality compared to those without diabetes [11]. According to a meta-analysis, diabetes is linked to a higher risk of mortality from vascular diseases such as coronary heart disease and ischemic stroke [12]. The current treatment of T2DM disease patients is based on drugs such as metformin, sulfonylureas, dipeptidyl peptidase-4 (DPP-4) inhibitors, sodium–glucose cotransporter 2 (SGLT2) inhibitors, glucagon-like peptide-1 (GLP-1) receptor agonists, and thiazolidinediones, which work to lower blood sugar levels by increasing the sensitivity of the body to insulin but are associated with side effects. In addition to iminosugars, Glyset^®^ was approved for the treatment of T2DM due to its ability to limit glucose absorption from the gut via intestinal α-1,4-glucosidase inhibition, lowering carbohydrate breakdown in the upper gastrointestinal tract. Unfortunately, the commencement of adverse effects occurs primarily at the level of the digestive system, where undigested saccharides provide a food supply for microbial fermentation [13].

To combat T2DM or even prevent it by delaying and/or halting the progression of the disease and the deterioration in its early stages, new natural-source-derived medications are urgently needed. These medications could lessen the side effects of currently prescribed medications and promote healthy ageing.

In recent years, nutritional inadequacies in the diet have been established as the key risk factor for T2DM [14,15,16,17,18]. Additionally, nutritional therapies have been shown to be successful in the treatment and prevention of chronic illnesses without having any negative side effects [19,20]. Unfortunately, food supplements high in amino acids (AA) and minerals are expensive; therefore, ingesting foods rich in nutrients, such as animal byproducts, is a low-cost approach to addressing this disease [21,22]. Bone broth is one of the numerous nutrient-dense animals-derived foods. Long-term bone boiling has been shown to produce a high concentration of AA, minerals, and proteins such as collagen. Scientific approaches have not been employed to completely study the medicinal qualities of this meal. However, it is ingested in countries like Mongolia to activate the immune system and support appropriate digestive system functions, with these therapeutic benefits attributable to its nutritional composition [23,24,25].

The nutritional composition of bones from bovine (12 months), chicken (4 months), sheep (13 months), and goat (9 months), as well as their biological activities, particularly those connected to T2DM, have not yet been documented in the literature. Therefore, the objective of the current investigation was to evaluate the nutritional profile of femur bones from bovine, chicken, sheep, and goats, as well as the possible therapeutic and positive effects of these extracts on the regression of T2DM in a Wistar albino rat model.

## 2. Materials and Methods

### 2.1. Chemicals and Reagents

The El-Nasr Company for Pharmaceuticals and Chemicals, Cairo, Egypt, supplied the methanol (MeOH), ethanol (EtOH), *n*-hexane, sulfuric acid, hydrochloric acid (HCl), nitric acid (HNO_3_), and sodium bicarbonate (NaHCO_3_). Additionally, Biosystems-SA, Costa-Brava 30, Barcelona (Spain), and DiaSys-Diagnostic-Systems-GmbH, Holzheim, Germany, provided all the kits needed for this biological study.

### 2.2. Preparation of the Bone Extract

The fresh bone extracts were made from bovine (1 kg, 12 months), chicken (1 kg, 4 months), sheep (1 kg, 13 months), and goat (1 kg, 9 months) femur bones, separately. The bones were purchased commercially and painstakingly sliced crosswise to produce chunks weighing between 100 and 130 g. The bones were cleaned in distilled water at 50 °C for 15 min before being discarded to eliminate any leftover meat, fat, or blood. This technique was performed three times. Each extract was prepared separately in a slow cooker (Taurus, Oliana, Spain). The bones used in the preparation were weighed at a 1:4 weight ratio. The extract was boiled in acidified water, which was created by combining 1 L of distilled water with 20 mL of white vinegar. Before incorporating the bones, the water was heated to boiling (100 °C). The cooking temperature was maintained at 100 ± 2 °C for 24 h. To maintain the initial volume of the solution, acidified distilled water was added. The obtained extracts were concentrated and dried in a rotary evaporator (Buchi Rotavapor R-300, Cole-Parmer, Vernon Hills, IL, USA) under vacuum at 70 °C to yield 30, 25, 20, and 16 g of each of the respective crude extract, and they were kept in a −20 °C freezer for future study [26,27].

### 2.3. Proximate Analysis

Crude protein was assessed by using a BCA Protein Assay Kit [28], crude fat was determined by solvent extraction (Method 991.36), and moisture was obtained by oven drying (Method 950.46) [28]. This technique was carried out in triplicate.

### 2.4. FAME Preparation

We performed the methylation in accordance with Alsenani et al., 2021 [29]. In 1 mL of *n*-hexane, 5 mg of each extract was suspended separately. The vials were sealed after being filled with a 2 mL aliquot of methanolic sulfuric acid (1%, *v*/*v*). The sample was heated at 50 °C in a stopper tube for 16 h. To halt the procedure, 2 mL of aqueous sodium bicarbonate (2% *w*/*v*) was added. The products were then extracted by using *n*-hexane (2–5 mL). In a rotary evaporator (Buchi Rotavapor R-300, Cole-Parmer, Vernon Hills, IL, USA) under vacuum and at 40 °C, the samples were concentrated and dried in the end [29,30,31,32].

### 2.5. FAME GC-MS Analysis

Gas chromatography–mass spectrometry (GC/MS) was used to analyze the recovered FAME extracts individually [31]. The TRACE GC Ultra Gas Chromatograph (Thermo Scientific Corp., Waltham, MA, USA) was used in conjunction with an ISQ Single Quadrupole Mass Spectrometer (Thermo Fisher Scientific, Waltham, MA, USA) as the detector of the instrument, and it featured the following specs: A TR-5 MS column (30 m × 0.32 mm i.d., 0.25 μm film thickness) was installed in the GC-MS system. The following temperature program was used for the analyses, which used helium as the carrier gas at a flow rate of 1.0 mL/min and a split ratio of 1:10: 60 °C for 1 min, followed by a 4.0 °C/min ascent to 240 °C and a 1 min hold. At 210 °C, the injector and detector were maintained. There was always a 1 μL injection of diluted samples of the combinations (1:10 *n*-hexane, *v*/*v*). By using a spectral range of *m*/*z* 40–450 and electron ionization (EI) at 70 eV, mass spectra were produced. Using the AMDIS program (www.amdis.net, accessed on 24 May 2022), the chemical components of the fatty acids were deconvoluted and identified by their retention indices in relation to the *n*-alkanes C8–C22 [31,32,33].

### 2.6. Analysis of Amino Acids

The Sykam-Amino-Acid Analyzer (Sykam GmbH, Eresing, Germany) equipped with Solvent-Delivery-System-S 2100 (a quaternary pump with a flow range of 0.01 to 10.00 mL/min, and a 400 bar maximum pressure was possible), Autosampler-S 5200, Amino Acid Reaction Module-S4300 (having a consistent signal output, a dual-filter photometer between 440 and 570 nm, and a signal summary option), and Refrigerated Reagent Organizer-S 4130 were used. To prepare a standard, a stock solution of ammonia and 18 amino acids (aspartic acid, serine, threonine, proline, glycine, glutamic acid, alanine, valine, cystine, methionine, leucine, isoleucine, tyrosine, histidine, phenylalanine, lysine, and arginine) were used. The quantities of all the amino acids were 2.5 µMol/mL (except for cystine with 1.25 µMol/mL), and then the standard was diluted to 60 µL in a 1.5 mL vial by using a sample dilution buffer whose composition is presented in Table 1. The mixture was then filtered by using a 0.22 µm syringe filter, and then an aliquot of 100 µL was injected. For sample preparation, 5 mL of *n*-hexane and 300 mg of the sample were combined. The mixture was then allowed to macerate for 24 h. The combination was filtered by using Whatman No. 1 filter paper, and the remnant of the filtrate was put into a test tube and was exposed to heat in an oven with 10 mL of 6N HCl for 24 h at 110 °C. After incubation, the material was filtered by using Whatman No. 1 filter paper and evaporated by using a rotary evaporator (Buchi Rotavapor R-300, Cole-Parmer, Vernon Hills, IL, USA) under vacuum at 40 °C and then dissolved completely in a 100 mL dilution buffer (Table 1). Then, 1 mL was diluted up to 3 mL by using a dilution buffer, filtered through a 0.22 µm syringe filter, and 100 µL was injected. The following instrument parameters were used: column; LCA K06/Na; and mobile phase: buffer A and buffer B (Table 1). For the regeneration solution mode of elution, a gradient was applied with a flow rate of 0.45 mL/min. The temperature followed a gradient of 57–74 °C, and the wavelengths of the buffers were 440 and 570 nm (Table 1). This technique was carried out in triplicate.

### 2.7. Mineral Analysis

To create powders with small particle sizes, samples were mashed with a mortar and pestle and sieved through meshes of various sizes (0.25, 0.212, and 0.16 mm). Wet digestion was used to prepare the samples. For sample digestion, aqua regia (a mixture of conc. HNO_3_ and HCl in a 1:3 ratio) was used. In a 100 mL glass flask, 1 g of each dry powdered sample was properly weighed and combined with 7 mL of HNO_3_ and 21 mL of HCl. The mixture was placed on a hot plate at 120 °C for around 5 h. After cooling, the samples were filtered by using filter paper (Whatman^TM^, Kent, UK), diluted to approximately 100 mL, and analyzed by ICP-OES. To avoid contamination, all the glassware, including the sample bottles and pipettes, were washed, disinfected, and rinsed with diluted HNO_3_, followed by distilled water. The samples were analyzed by using Ultima Expert LT ICP-OES (HORIBA Scientific, HORIBA France SAS, Longjumeau, France) [34,35] to determine aluminum (Al), cadmium (Cd), lead (Pb), chromium (Cr), calcium (Ca), cobalt (Co), copper (Cu), iron (Fe), magnesium (Mg), phosphorus (P), and zinc (Zn) with the parameters shown in Table 2. This technique was performed three times.

### 2.8. Animals

The Animal House of the Faculty of Pharmacy at Beni Suef University provided the male Wistar albino rats (130 ± 10 g), which were kept in groups of 10 rats per cage and maintained in a controlled environment at 26–29 °C. They were provided with a fixed light/dark cycle for 1 week as an adaptation period to acclimatize under a normal combination with free access to water and food.

#### 2.8.1. Animal Ethical Statement

This study was approved by the Ethical Committee of Beni Suef University, Egypt, provided that the animals will not suffer at any stage of the experiment and be maintained in accordance with the guide for the care and use of laboratory animals (ethical approval no: 022-369).

#### 2.8.2. Acute Toxicity Study

Serial concentrations of 500–2000 mg/kg b.wt. of each extract were tested for the determination of the LD_50_ value. In an acute toxicity study of extracts 1–4, a total of 48 rats (four rats for each dose) were used in this study and observed for 24 h. No mortality and no toxicity signs were observed up to 2000 mg/kg b.wt. over 24 h. The selected dose used was 500 mg/kg b.wt. [36].

#### 2.8.3. In Vitro DPPH Radical Scavenging Activity Assay of the Investigated Bone Extracts

The stable radical 2,2-diphenyl-1-picrylhydrazyl (DPPH) assay was used to investigate the radical scavenging capacity of femur bone extracts from bovine (12 months), chicken (4 months), sheep (13 months), and goat (9 months) [37]. In a quick procedure, 2 mL of the freshly made DPPH solution (20 g/mL in absolute ethanol) were combined with 1 mL of the tested extract at various concentrations (0.01, 0.05, and 0.1 μg/mL in absolute ethanol), and then the mixture was incubated at room temperature in the dark for 30 min. A UV-Vis Jenway 6300 spectrophotometer (Jenway, Vietnam, UK) was used to measure the absorbance at λ517 (nm). Absolute ethanol served as a blank while ascorbic acid served as a positive control. The following equation was used to compute the DPPH radical scavenging activity:

$$\%\;DPPH\;scavenger\;activity\;=\frac{\;absorbance\;ofblank\;-\;absorbance\;of\;tested\;sample}{absorbance\;of\;blank}\;\times \;100$$

#### 2.8.4. Induction of T2DM and Experimental Design

The induction of T2DM was performed through a single intraperitoneal injection of streptozotocin (STZ) (60 mg/kg b.wt., dissolved in 0.01 M citrate buffer, pH 4.5) [38,39]. Six hours after the STZ injection, the rats were orally given a 5% glucose solution for 24 h to avoid hypoglycemic shock. The animals were then subjected to routine observation in conventional settings. The fasting blood glucose level of the rat was measured on day 5 after the STZ injection by measurement of the tail vein blood glucose levels by using a portable glucometer (Accu-Chek Active, Roche Diagnostics Ltd., Mannheim, Germany). Again, all of the test animals were maintained under observation to see if the hyperglycemic status of diabetes-induced rats could be stabilized. Fasting blood glucose levels were measured again on day 10 after the STZ injection. Diabetes was defined as blood glucose levels greater than 200 mg/dL in STZ-injected rat.

The rats were put into seven groups of ten each. The rats in Group I were fed ordinary normal chow and received a single intraperitoneal injection of 0.01 M citrate buffer (pH 4.5). The Group 2 (STZ-diabetic group) rats received a single intraperitoneal STZ injection (150 mg/kg b.wt., dissolved in 0.01 M citrate buffer, pH 4.5) but received no other therapy. The rats in Groups 3–6 (diabetic rats treated with bovine (12 months), chicken (4 months), sheep (13 months), and goat (9 months) femur bone extracts, respectively) received a daily oral dose of 500 mg/kg b.wt. of their respective extracts. Group 7 consisted of diabetic rats treated with a daily oral dose of the reference drugs glibenclamide (10 mg/kg b.wt.) [40].

#### 2.8.5. Sample Preparation

After one month of the appropriate food treatment, the diabetic rats were given blood samples by rupturing the sublingual vein after being fasted overnight, mildly sedated with thiopental (30 mg/kg b.wt.), and lightly anaesthetized. The serum was created through centrifugation at 3000 rpm for 15 min at 4 °C and used for a biochemical analysis of lipid profiles, inflammatory markers, liver function, and kidney function. After that, cervical dislocation was used to sacrifice the rats. The livers were rapidly removed, cleaned in saline, dried on filter paper, and homogenized in a 50 mM phosphate buffer, pH 7.4, by using an Ultra-Turrax homogenizer. The resulting homogenate (20% *w*/*v*) was then centrifuged at 3000 rpm for 15 min at 4 °C. The resulting supernatant was kept at −80 °C for further research.

#### 2.8.6. Estimation of Blood Glucose Profiles Parameters

The blood glucose levels were measured by using a portable glucometer (Accu-Chek Active, Roche Diagnostics Ltd., Mannheim, Germany) in the rats’ tail vein on days 0, 5, 10, and 28 of diabetes induction.

#### 2.8.7. Estimation of Lipid Profile Parameters

Using kits provided by Biodiagnostics (Cairo, Egypt), lipid profile assessments were carried out by estimating the serum TC, HDL-C, TG, and TL in accordance with the methods of Richmond, 1973 [41]; Wieland and Seidel, 1983 [42]; Burstein et al. 1970 [43]; Fossati and Prencipe, 1982 [44]; and Connerty et al. 1961 [45].

#### 2.8.8. Estimation of Hepatic Functions Markers

Investigations of the serum AST, ALT, ALP, and total bilirubin were conducted according to Elmaidomy et al. 2020 [46], by using kits provided from Biodiagnostics (Egypt). The total protein was estimated according to Gornall et al. 1949 [47].

#### 2.8.9. Estimation of Kidney Functions Markers

Using kits acquired from Biodiagnostics (Egypt), the serum urea was measured by using colorimetric techniques in accordance with the Fawcett and Scott method [48], while serum creatinine was measured by using kits acquired from Biodiagnostics, Egypt, following the kinetic analysis method described by Bartels et al. 1972 [49]. According to Fawcett and Scott, 1960 [50], the urea level was calculated.

#### 2.8.10. Estimation of Proinflammatory Markers

An enzyme-linked immunoassay (ELISA) kit bought from Quantikine^®^, R&D Systems (Minneapolis, MN, USA) was used to determine the levels of serum TNF- and IL-6. An ELISA kit was used to determine the levels of sICAM-1 and sVCAM-1, and it was purchased from Quantikine^®^, R&D Systems (MN, USA) and EIAab^®^ (College Park, MD, USA), respectively.

#### 2.8.11. Estimation of Oxidative Stress Markers

Using kits obtained from Biodiagnostics (Egypt), the oxidative stress markers hepatic MDA and GSH were calculated in accordance with Elmaidomy et al. 2020 [46].

#### 2.8.12. Histopathological Investigation

Liver and pancreas samples were preserved in 10% formalin, while pancreas samples were preserved in Bouin’s solution. The samples were molded into paraffin beeswax tissue blocks and cut into 4 μm thick sections and stained with a hematoxylin and eosin (H&E) stain for routine examination under an electric light microscope [51,52].

#### 2.8.13. Histopathological Lesion Scoring

Histopathological alterations in the liver and pancreas were recorded and scored as: no changes (0), mild (1), moderate (2), and severe (3) changes; the grading was determined as follows: <30% changes (mild change), changes in a range from 30 to 50% (moderate change), and >50% changes (severe change) [53].

### 2.9. Statistical Analysis

Data are expressed as mean ± standard deviation (SD). A one-way analysis of variance (ANOVA) was used to statistically analyze the data, and then a post hoc and costate computer program was used to compare the means of the therapy groups. Different letters were considered significant at *p* ≤ 0.05 by using SPSS (SPSS for Windows 7, version 8, Chicago, IL, USA) software.

$$\%\;change=\frac{mean\;of\;negative\;control-mean\;of\;treatment\;group}{mean\;of\;negative\;control}\times 100,$$

$$\%\;improvment=\frac{mean\;of\;positive\;control-mean\;of\;treatment\;group}{mean\;of\;negative\;control}\times 100$$

## 3. Results

### 3.1. Proximate Composition

Table 3 shows the proximate compositions of bovine (12 months), chicken (4 months), sheep (13 months), and goat (9 months) femur bone extracts, whereas the moisture contents of the goat and chicken bones were discovered to be higher than those of the bovine and sheep bones (Table 3), with a significant difference (*p* ≤ 0.05). Chicken (11,000 ± 0.002) and goats (10,000 ± 0.001) had the lowest crude protein content, whereas sheep (16,000 ± 0.003) and bovine (17,000 ± 0.008) had higher levels (*p* ≤ 0.05). The crude fat content was, however, lower in chicken (0.120 ± 0.008) and goat (0.100 ± 0.004) and higher in bovine (0.170 ± 0.006) and sheep (0.160 ± 0.005), both with a *p*-value of ≤0.05.

### 3.2. Fatty Acid Composition

The GC-MS analysis identified a total of ten compounds, representing 99.96% of the total detected peaks for the fat content of bovine femur bone extract (Table 4, Figure S1). These identified compounds belonged to the fatty acid chemical class (Table 4). As demonstrated in Table 4, the detected fatty acids ranged from monounsaturated fatty acids (two MUFA, 27.25%) to polyunsaturated fatty acids (one PUFA, 0.80%), with saturated fatty acids (seven SFA, 71.91%) being the main fatty acid fraction. Among the SFAs, eicosanoic, palmitic, and pentadecanoic acids were the most abundant, making up almost 25.70%, 19.30%, and 15.94% of the total fatty acids (FA), respectively (Table 4). Among the unsaturated fatty acids (USFA), oleic acid was the most abundant MUFA, making up almost 22.99% of the total MUFAs.

While the GC-MS analysis identified a total of 18 compounds representing 99.83% of the total detected peaks for the fat content of the femur bone extract of the chicken (Table 4, Figure S2), which were found to belong to the fatty acid and sterol chemical classes (Table 4), the identified fatty acids also varied from SFAs (seven SFAs, 41.47%) to MUFAs (2 MUFA, 55.52%), representing the major fatty acid fraction, and PUFAs (seven PUFAs, 2.58%), as shown in Table 4. Among the SFAs, palmitic acid was the most abundant, making up almost 33.89% of the total FAs (Table 4). Among the USFAs, oleic acid was the most abundant MUFA, making up almost 54.70% of the total MUFAs. Combined *n*-3 PUFAs (C18:2, C18:2, C18:3, C17:3, C18:3, C20:4, and C18:2) accounted for 2.58% of the total FAs in the femur bone extract of the chicken concerning the likewise present long-chain fatty acids (Table 4).

Regarding the fat content of the sheep femur bone extract (Table 4, Figure S3), the GC-MS analysis identified a total of 15 compounds, representing 98.88% of the total detected peaks. These identified compounds belonged to the chemical class of fatty acids and sterols (Table 4). The identified fatty acids varied from SFAs (eight SFAs, 42.31%) to MUFAs (three MUFAs, 56.46%), representing the major fatty acid fraction, and PUFAs (three PUFAs, 1.96%), as shown in Table 4. Among the SFAs, palmitic acid was the most abundant one, making up almost 23.62% of the total FAs (Table 4). Among the USFAs, oleic acid was the most abundant MUFA, making up almost 53.80% of the total MUFAs.

And regarding the goat femur extract (Table 4, Figure S4), the GC-MS analysis identified a total of 11 compounds, representing 94.67% of the total detected peaks. These identified compounds belonged to the fatty acid and sterol chemical classes too (Table 4). The identified fatty acids varied from SFAs (two SFAs, 29.2%) to MUFAs (three MUFAs, 63.48%), representing the major fatty acid fraction, and PUFAs (three PUFAs, 0.73%), as shown in Table 4. Among the SFAs, palmitic, stearic, and myristic acids were the most abundant ones, making up almost 12.07%, 9.03%, and 7.89%, respectively, of the total FAs (Table 4). Among the USFAs, oleic acid was the most abundant MUFA, making up almost 60.81% of the total MUFAs.

Comparing the GC-MS analysis results, the SFA content of the bovine femur extract (71.91%) was the highest, followed by chicken (41.47%), sheep (42.31%), and goats (29.2%). Palmitic acid (12.07–33.89%) and oleic acid (22.99–60.81%) were the two most abundant fatty acids in the bovine (12 months), chicken (4 months), sheep (13 months), and goat (9 months) femur bone extracts. Only the chicken, sheep, and goat femur bone extracts contained myristic acid (0.86–7.89%) and stearic acid (0.13–9.03%). In the bovine, chicken, and sheep femur extracts, palmitoleic acid (0.35–4.26%) and eicosanoic acid (0.84–25.70%) predominated (Table 4).

SFA consumption has been reduced in dietary recommendations to reduce the risk of CVD, while the 2015–2020 Dietary Guidelines for Americans recommend replacing SFAs with both MUFAs and PUFAs to lower CVD risk [54]. According to the results of our GC-MS analysis, the bone extract of the goat possessed the most necessary fatty acids (64.21%), which were represented as C18:1 (9), C18:2 (6, 9), C18:3 (9, 12, 15), C20:1 (11), and C17:1 (6). Table 4 shows that chicken and sheep had similar essential fatty acids (58.10% and 56.46%, respectively), expressed as C16-20:1-4.

### 3.3. Amino Acid Content

The amino acid content of the bovine (12 months), chicken (4 months), sheep (13 months), and goat (9 months) femur extracts are presented in Table 5, Figures S5–S8. The four extracts were rich in proline (0.873–3.570 mg/100 mg), glycine (0.714–4.254 mg/100 mg), alanine (0.458–3.370 mg/100 mg), and arginine (0.168–1.983 mg/100 mg). According to the literature, glycine and proline are the major components of collagen; additionally, proline elicits stress-stimulated phenolic biosynthesis and stimulates antioxidant enzyme response pathways [55].

The amino acid content of the goat bone extract (18.708 mg/100 mg) was higher than that of the chicken (11.753 mg/100 mg, 4 months), bovine (7.237 mg/100 mg, 12 months), and sheep (3.823 mg/100 mg, 13 months). In comparison to the bovine (12 months), chicken (4 months), and sheep (13 months), the goat femur bone extract had the highest concentration of essential amino acids, including threonine, valine, isoleucine, leucine, phenylalanine, histidine, and lysine (0.235, 0.701, 0.366, 1.284, 0.623, 0.522, and 0.978 mg/100 mg, respectively); see Table 5.

Amino acids are required for the synthesis of many proteins that provide critical activities, such as carriers of carbon dioxide (CO_2_), oxygen (O_2_), structural proteins, and vitamins [1]. The goat femur extract appears to be an important source of high-biological-value proteins due to its essential amino acid content (Table 5).

### 3.4. Mineral Contents

The mineral makeup of the femur bone extracts from bovine (12 months), chicken (4 months), sheep (13 months), and goat (9 months) are displayed in Table 6. All elements were extracted in greater quantities after adding acid to the broth, with the exception of Fe and Zn, according to the literature (Table 6). However, increases in the hazardous metals Pb, Cd, Cr, and Al were smaller. In addition, the four extracts had larger concentrations of Ca, Mg, Zn, P, and Fe, which suggests that they may greatly increase the daily intake of these crucial minerals (Table 6).

These extracts contain dangerous metal concentrations per serving that range from a few μg (Cd) to hundreds of μg (Al, Pb). These concentrations are below the recommended maximum tolerated daily intake (PMTDI) dosage, which leads to minimal danger concentrations [23]. As a result, it is believed that there is no interaction between the harmful metals present in one serving of these extracts, and the health risks associated with ingesting them are minor (Table 6). However, consuming a lot of long-cooked bone broth is not advised because it could contain a lot of oil-based components like vitamin D, which comes from fatty bone marrow and can cause hypercalcemia if taken in excess [23].

### 3.5. In Vitro DPPH Radical Scavenging Activity Assay of Bovine (12 Months), Chicken (4 Months), Sheep (13 Months), and Goat (9 Months) Femur Bone Extracts

The extreme scavenging behavior of the bovine (12 months), chicken (4 months), sheep (13 months), and goat (9 months) bone extracts were tested by using the stable-radical DPPH-assay. The results revealed a considerable increase in the DPPH scavenging activity as the extract concentration increased (a dose-dependent relationship). The highest DPPH scavenging activity was demonstrated by the bovine extract, which was followed by the sheep extract, goat extract, and finally chicken extract (Table 7).

### 3.6. Estimation of Blood Glucose Profiles Parameters

Blood sugar levels in the diabetic rats increased significantly, reaching 218.18% when compared to the controls. The diabetic rats treated with the bovine (12 months), chicken (4 months), sheep (13 months), and goat (9 months) femur bone extracts showed a significant improvement with the bovine (177.30%), sheep (172.73%), goat (159.10%), and finally chicken extract (155.50%) when compared to the standard drug, which showed a degree of improvement of 190.91% (Table 8).

### 3.7. Estimation of Lipid Profiles Parameters

Significant increases in the TL, TG, and TC occurred, while a significant reduction in the HDL-C with percentages of 108.33, 145.10, 153.64, and 61.80% occurred. In the diabetic rats, oral treatment of diverse extracts successfully improved the lipid profile, as demonstrated by a considerable reduction in the TL, TG, and TC levels, with improvements of 98.33, 68.63, and 114.55%, respectively, with the bovine extract (*p* ≤ 0.05), while there was a 24% increase in the serum HDL-c level. Meanwhile, the oral treatment of the diabetic rats with sheep extract also efficiently decreased the serum TL, TG, and TC with improvements of 95.00, 63.73, and 100.00%, respectively, while significantly increasing the HDL-C levels with improvements of 17.80, followed by goat extract and finally chicken extract compared with the standard drug, which showed ameliorated values of 100.00, 136.07, 117.27, and 35.80% for the TL, TG, TC, and HDL-C, respectively (Table 9).

### 3.8. Estimation of Hepatic Functions Markers

Diabetes has a significant impact on numerous endogenous organs, the liver being one of the most critical [56]. Hyperglycemia-induced oxidative stress and the consequent abnormalities in glucose, protein, and lipid metabolisms are the most prominent causes of diabetic liver injury [57]. As demonstrated in Table 10, the diabetic group experienced increased ALT, AST, ALP, and bilirubin levels by 170.56%, 84.03%, 111.11%, and 159.30%, respectively, compared to the control group (*p* ≤ 0.05), while the total protein levels decreased by 62.96%. In comparison to the diabetic rats, the oral administration of the bovine (12 months), chicken (4 months), sheep (13 months), and goat (9 months) bone extracts successfully ameliorated liver function, manifested as an effective reduction for the bovine extract in ALT, AST, ALP, and bilirubin and an improvement of 94.12%, 69.44%, 55.56%, and 92.59%, respectively (*p* ≤ 0.05), conveyed by a significant increase in the serum total protein level by 36.67%. The results of the bovine extract are followed by those of the sheep, goat, and finally chicken extracts compared to the standard (Table 10).

### 3.9. Estimation of Kidney Functions Markers

As indicated in Table 11, the creatinine and urea levels of the diabetic group increased by 132.35% and 191.18%, respectively (*p* ≤ 0.05), as compared to the control group. Compared to the diabetic rats, the oral administration of the bovine (12 months), chicken (4 months), sheep (13 months), and goat (9 months) bone extracts efficiently enhance renal function, as seen by a notable decrease in creatinine and urea and improvements of 85.29% and 144.12%, respectively (*p* ≤ 0.05), with the bovine extract, while the improvement reached 67.65% and 138.24%, respectively, with the sheep extract compared to the standard drug (117.65% and 164.71% for creatinine and total urea, respectively). Extracting goat and chicken showed improvements of 55.88% and 88.23% for the goat and 44.12 and 79.41% for the chicken extract regarding creatinine and urea, respectively (Table 11).

### 3.10. Estimation of Proinflammatory Markers

When compared to the control values, the diabetic control rats showed a considerable rise in serum TNF-α and IL-6 levels of 168.69% and 118.18%, respectively. The treatment of the STZ-induced diabetes with the bovine extract showed an improvement of 76.77% and 41.82% for TNF-α and IL-6, respectively, while the improvement with the sheep extract reached 73.74 and 95.45%, respectively. These results are followed by the goat extract and finally chicken extract, where therapy with the goat extract demonstrated a significant decrease in serum TNF- and IL-6, with improvements of 66.67 and 40.91%, respectively. Meanwhile, the values recorded were 73.74% and 95.45%, respectively, for the treatment with the chicken extract compared to the standard drug (147.47% and 90.91%, respectively, for TNF-α and IL-6) (Table 12).

The STZ-induced diabetes rats suffered from a significant increase in sICAM-1 and sVCAM-1, as demonstrated in Table 12. This increase reached 63.64% and 1725%, respectively, compared to normal control levels. Treatment with the bovine extract and sheep extract caused significant reductions in the sICAM-1 and sVCAM-1 levels, with the highest degrees of improvement (181.82 and 950.00%, respectively) occurring with the bovine extract, while the sheep extract resulted in improvements of 151.52% and 750.00%, respectively. A noticeable improvement was also recorded upon the treatment of diabetic rats with the goat and chicken extracts, where they exhibited a marked amelioration of sICAM-1 and sVCAM-1 levels compared to when standard drugs were used (Table 12).

### 3.11. Estimation of Oxidative Stress Markers

Untreated diabetic rats had a considerable increase in hepatic MDA, reaching a value of 309.10%. However, when compared to the normal control, there was a considerable decline in hepatic GSH of 73.33%. The treatment of STZ-induced diabetic rats with bovine extract showed the highest improvement in MDA reduction and a noticeable elevation in GSH levels by 227.27% and 42.67% for MDA and GSH, respectively. This was followed by sheep extract (200% and 41.33% for MDA and GSH, respectively), goat extract, and finally chicken compared to standard drugs, which exhibited a significant reduction in MDA by 300.91% and a significant increase in GSH by 49.33%, as shown in Table 13.

### 3.12. Histopathological Results

#### 3.12.1. Histopathological Results of Liver

**Figure 1:**
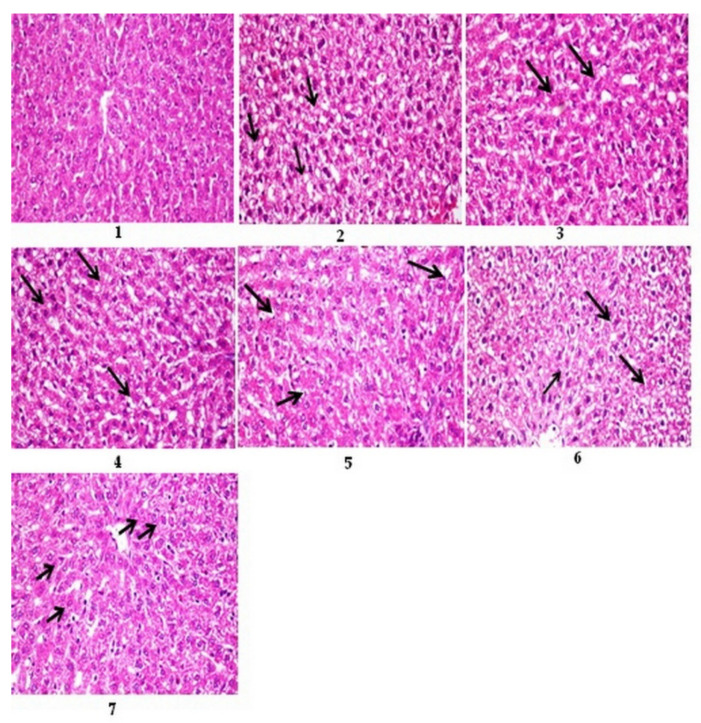
Photomicrographs of sections of rats’ liver stained with H&E 400. Photo 1: control rat liver, showing normal histological structure of the central vein and hepatocytes. Photo 2: diabetic rat liver showing macrovesicular steatosis of hepatocytes (arrows). Photo 3: diabetic rat liver treated with bovine extract showed mild vacuolar degeneration of hepatocytes (arrow). Photo 4: diabetic rat liver treated with chicken extract, showing mild steatosis of hepatocytes (arrow). Photo 5: diabetic rat liver treated with sheep extract, showing mild vacuolar degeneration in a few hepatocytes (arrow). Photo 6: diabetic rat liver treated with goat extract shows severe vacuolar degeneration of hepatocytes (arrows). Photo 7: diabetic rat liver treated with the standard drug glibenclamide showed mild vacuolar degeneration of hepatocytes (arrow).

The livers of diabetic rats showed macrovesicular steatosis of hepatocytes compared to the control (Figure 1, Photos 1 and 2). However, the diabetic rat liver treated with the bovine (12 months), chicken (4 months), sheep (13 months), and goat (9 months) femur extracts or with the standard drug displayed mild steatosis and vacuolar degeneration of fewer hepatocytes compared to the standard drug (Figure 1, Photos 3–7).

#### 3.12.2. Histopathological Results of Pancreas

**Figure 2:**
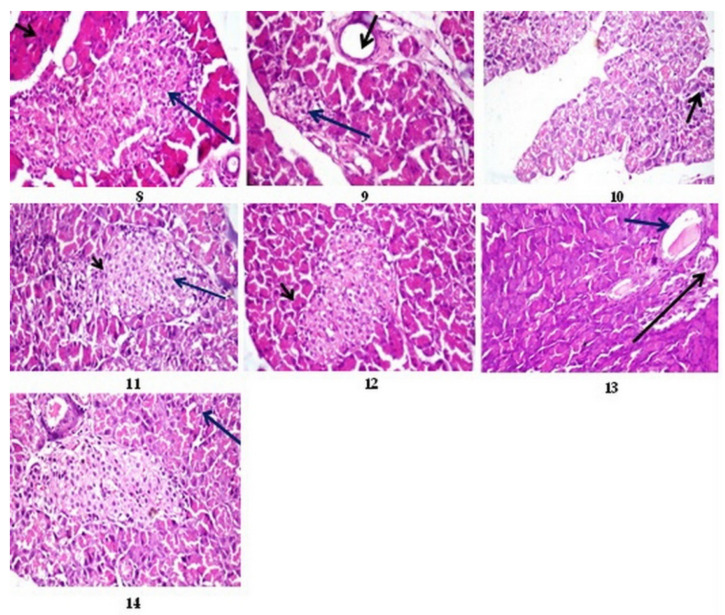
Photomicrographs of sections of pancreatic rats stained with H&E 400: Photo 8: control rat pancreas, showing normal histological structures of Langerhans islets (long arrow) and exocrine pancreas (short arrow). Photo 9: diabetic rat pancreas, showing atrophy and vacuolation of Langerhans islets (long arrow) with periductal fibrosis and ductal dilatation (short arrow). Photo 10: diabetic rat pancreas treated with bovine extract shows vacuolar degeneration of the lining epithelium of the exocrine pancreas (arrow). Photo 11: diabetic rat pancreas of rats treated with chicken extract, showing nearly normal size of Langerhans islets and normal exocrine pancreas with mild vacuolar degeneration in cells of Langerhans islets (arrow). Photo 12: diabetic rat pancreas of rats treated with sheep extract, showing normal islets of Langerhans (long arrow) and exocrine pancreas (short arrow). Photo 13: diabetic rat pancreas of rats treated with goat extract, showing atrophy and distortion of Langerhans islets (long arrow) with ductal dilatation (short arrow). Photo 14: diabetic rat pancreas of rats treated with standard drugs show normal-sized islets of Langerhans with vacuolation and mild congestion (arrow).

According to Figure 2, Photo 9, the diabetic rat pancreas exhibits vacuolar degeneration and necrosis of the exocrine pancreas, interstitial congestion, edema, and hemorrhage, as well as distortion and atrophy of the islets of Langerhans compared to the control (Photo 8). The diabetic rat pancreas treated with the bovine (12 months), chicken (4 months), sheep (13 months), and goat (9 months) bone extracts as well as standard drugs, on the contrary, showed vacuolation and mild congestion (arrow) vacuolar degeneration of the lining epithelium of the exocrine pancreas, nearly the normal size of the Langerhans islets, and normal exocrine pancreas with mild vacuolar degeneration in the cells of the Langerhans islets (H&E 400) (Photos 10–14).

#### 3.12.3. Histopathological Lesion Scoring

The score system was designed as follows: score 0 = absence of the lesion in all rats in the group (n = 5); score 1 (<30%); score 2 (30–50%); score 3 (>50%); G1: control; G2: diabetes; G3–G6: diabetics treated with bovine (12 months), chicken (4 months), sheep (13 months), and goat (9 months) femur extracts, respectively; and G7: diabetics treated with standard drugs. The diabetic rats treated with the bovine extract showed the lowest level of lesion scores, which confirmed the biochemical results, followed by the rats treated with the sheep extract, goat extract, and finally chicken extract (Table 14).

## 4. Discussion

Diabetes mellitus is a chronic metabolic disease characterized by elevated blood glucose levels which, over time, causes damage to the heart, kidneys, vasculature, eyes, and nerves [58]. More than 90% of cases of diabetes mellitus are T2DM, which is distinguished by tissue insulin resistance (IR), an insufficient compensatory insulin secretory response, and insufficient insulin production by pancreatic islet cells [58,59]. As the disease progresses, insulin secretion is unable to maintain glucose homeostasis, resulting in hyperglycemia [59]. Patients with T2DM are typically obese or have a higher body fat percentage. Adipose tissue promotes IR in this condition via a variety of inflammatory mechanisms, including increased free fatty acid (FFA) release and adipokine deregulation [60,61]. The pancreas (β-cells and α-cells), liver, skeletal muscle, kidneys, brain, small intestine, and adipose tissue are all involved in the development of T2DM [62]. Adipokine dysregulation, inflammation, and abnormalities in the gut microbiota have emerged as important pathophysiological factors [63]. The current treatment of T2DM patients is based on drugs that work to lower blood sugar levels by increasing the sensitivity of the body to insulin but are associated with side effects. There is a high demand for the discovery of new natural-source drugs aiming to protect against T2DM.

During the past years, nutritional deficiencies in the diet have been identified as the primary risk factor for T2DM [14,15,16,17,18]. Nutritional therapies have furthermore demonstrated efficacy in the treatment and prevention of chronic illnesses without producing negative side effects [19,20]. Unfortunately, food supplements rich in long-chain FA, AA, and minerals are costly and not a profitable solution; thus, ingesting nutrient-rich foods such as animal byproducts is a low-cost approach to combating the disease [21,22].

The present study therefore investigated the composition of bovine (12 months), chicken (4 months), sheep (13 months), and goat (9 months) femur extracts, including the fatty acids, amino acids, and minerals. Kim et al. 2017 [24], investigated the effects of slaughter age (28, 32, or 38 months) on the proximate composition, collagen content, fatty acid composition, amino acid content, and mineral contents of horse leg bone extracts (HLBE) derived from Jeju crossbred horses. The HLBE had higher levels of crude protein and collagen at 32 and 38 months than at 28 months. Palmitoleic acid and essential fatty acids were greater in the HLBP at 38 months versus 28 months. Except for histidine, nearly all amino acids were identified at higher amounts in the HLBP at 38 months than at 28 months. The HLBP’s P, K, Zn, Se, and Fe contents increased considerably with age. These findings imply that some nutrients in bone broths increase with age, and hence extracts would be more beneficial for human consumption.

Also, the present study evaluated the antidiabetic potential of the different extracts in Wistar albino rats in both in vitro and in vivo assays. The GC-MS analysis revealed the presence of a predominant percentage of long-chain fatty acids (myristic acid; pentadecanoic acid; palmitic acid; and isomargaric, oleic, eicosanoic, and stearic acids), which are reported to have protective activities against T2DM [64,65]. Free fatty acids function as signaling molecules in the secretion of insulin. A G-protein-coupled receptor (GPR40), which is highly expressed in the pancreas, serves as a receptor for long-chain FFAs [46], whereas long-chain FFAs trigger GPR40 to increase insulin production from pancreatic β cells.

Our amino acid analysis revealed the presence of a predominant percentage of essential amino acids, which are also reported to have repellent activities against T2DM [66,67,68,69,70]. Leucine is reported to act as a metabolic fuel and an allosteric activator of glutamate dehydrogenase to promote glutaminolysis in pancreatic β cells, where it abruptly enhances insulin production. At physiological doses, leucine has also been demonstrated to control gene transcription and protein synthesis in pancreatic islet β cells via both mTOR-dependent and -independent pathways. Leucine therapy has been found to alleviate the malfunctioning of the insulin secretory system in human diabetic islets by activating several vital metabolic genes [66]. Additionally, increased glucose absorption via the AMPK pathway by an APN-dependent mechanism was demonstrated by the evaluated proline, phenylalanine, and alanine’s antidiabetic effects in human visceral adipocytes in vitro [71].

On the other hand, bone extracts differ in their mineral content, e.g., Ca [72,73,74], Mg [75,76,77], and Zn [78], which have been linked to insulin secretion from β-cells, where Zn plays a key role in the insulin biosynthesis as part of the hexameric structure of this hormone and in the sensitivity to insulin in target tissues through the stimulation of insulin receptors [79]. Ca regulates voltage-dependent channels in pancreatic β-cells, which are essential for insulin exocytosis [80]. Mg is important for β-cell functioning and acts as a cofactor of many enzymes involved in glucose metabolism, like tyrosine kinase enzymes, which phosphorylate insulin receptors and trigger the signaling cascade [81].

The lipid peroxidation process may be considered a biomarker for diabetes through the activation of lipoxygenase enzymes [82], which contributed to the significant reduction in GSH and increase in MDA contents. Insulin resistance and adipose tissue hyperplasia, on the other hand, are considered inflammatory states that are associated with elevated proinflammatory mediators and cytokines, e.g., TNF-α and IL-6 [83]. Bone extracts differ in their mineral content, which affects oxidative stress and proinflammatory mediators and cytokines. For example, essential minerals such as Zn and Cu are recognized to play key roles in the maintenance of redox homeostasis, which is also required for the immune system. Changes in the status of these minerals may result in increased inflammatory responses and oxidative stress [84]. Disruptions in Zn homeostasis, for example, can cause a shift in the Th1/Th2 balance towards a Th2 response [85]. Additionally, Cu excess and deficiency can cause oxidative stress, which can result in chronic inflammation [86]. Elevated serum Cu may be a helpful indicator of immunological and inflammatory states [87].

In the current study, STZ was administered to male Wistar rats. As a reaction, the blood glucose levels increased significantly. While there was an improvement in the blood glucose levels after treating the diabetic rats with different bone extracts (bovine (12 months), chicken (4 months), sheep (13 months), and goat (9 months)), this can be explained by the extracts of fats, minerals, and proteins activating the pancreatic β-cells to compensate for and release insulin and stimulate insulin sensitivity to receptors, resulting in the activation of carbohydrate-metabolizing enzymes and thus the restoration of normal blood glucose levels (Table 8).

Also, the current study found that the diabetic rats had significantly higher levels of AST, ALT, and ALP enzymes. This increase has been linked to liver dysfunction caused by insulin deficiency syndrome [82]. The bilirubin level was also found to be elevated in diabetic rats (Table 9). Bilirubin is a cholestasis marker that can help protect against metabolic and cardiovascular diseases [82,88]. There was an improvement in AST, ALT, bilirubin, and ALP levels after treating the diabetic rats with different extracts (bovine, chicken, sheep, and goat bone extracts), particularly the bovine and sheep extracts.

Measuring urea and creatinine is often recognized as a biomarker for renal functioning since it can indicate diabetic nephropathy. The most severe side effects of diabetes include diabetic nephropathy and diabetic kidney disease (DKD) [89]. In terms of renal clearance performance and functions, an increase in serum urea and creatinine levels was seen in the untreated diabetes group (Table 10), while there was an improvement in the urea and creatinine levels after treating the diabetic rats with different extracts (bovine, chicken, sheep, and goat femur bone extracts), particularly in the case of the bovine and sheep extracts. Similarly, earlier reports have shown that naturally occurring amino acids have potent antioxidative activity in a wide variety of experimental systems and are clinically used to treat diabetic neuropathy [90,91].

Diabetes is also linked to hyperlipidemia [10]. In this investigation, diabetic rats had significantly higher levels of TG, TL, and TC than the control rats, although the HDL-C levels were significantly lower (Table 11). The altered lipid profile values in the diabetic rats may be attributed to the decreased activity of cholesterol biosynthesis enzymes or low levels of lipolysis controlled by insulin [92], while there was an improvement in the TG, TL, HDL-C, and TC levels when the diabetic rats were treated with different extracts (bovine, chicken, sheep, and goat bone extracts, Table 11).

Insulin resistance and adipose tissue hyperplasia, on the other hand, are considered inflammatory states that are associated with elevated proinflammatory mediators and cytokines [83]. In the current investigation, STZ-induced rats had significantly higher serum TNF-α and IL-6 levels (Table 12). The TNF-α and IL-6 levels improved when the diabetic rats were treated with various extracts, particularly bovine and sheep bone extracts.

CVD has been identified as a fatal result of T2DM [93]. In the current study, the untreated diabetes group had higher levels of adhesion molecules, which were attributed to enhanced phospholipase activity caused by increased vasoconstrictive eicosanoids [94]. However, the treatment of diabetic rats with various extracts (bovine, chicken, sheep, and goat femur bone extracts), particularly bovine and sheep extracts, resulted in a reduction in sICAM-1 and sVCAM-1 values, implying that these extracts may have mitigated the cardiovascular consequences of metabolic disorders to some extent (Table 12). Although amino acid shortages are known to occur in diabetic patients and are thought to contribute to the development of cardiomyopathy, the mechanisms of the influence of amino acid restoration on enhanced cardiac function are not fully understood. Diabetes was found to cause cardiac dysfunction, myocardial cell death, and alterations in plasma glucose and lipid levels. The treatment of diabetic rats with various amino acids reduced changes in heart function. Individually or in combination, the amino acids taurine, carnitine, and arginine reduced diabetes-induced cell damage. Carnitine alone lowered plasma TG levels while increasing HDL-C. These findings imply that dietary amino acid supplementation reduces diabetes-induced alterations in heart contractile function and ultrastructure [95].

Reactive oxygen species (ROS) are known to produce cellular and tissue injury through covalent binding, DNA strand breaking, lipid peroxidation, and augmenting fibrosis [96]. The lipid peroxidation process may be considered a biomarker for diabetes through the activation of lipoxygenase enzymes [82], which contributed to the significant reduction in GSH and increase in MDA contents. In the current study (Table 13), there was an improvement in GSH and MDA levels after treating the diabetic rats with different extracts (bovine, chicken, sheep, and goat bone extracts), particularly the bovine and sheep extracts. This can be explained by the high antioxidant scavenging activity of the extracts, especially the bovine bone and sheep extracts.

Histopathological observations confirmed the biochemical study, where the bovine and sheep extracts showed the best results, followed by the goat and chicken extracts (Table 14, Figure 1 and Figure 2).

## 5. Conclusions

The current study examined the nutritional composition of femur bone extracts from bovine (12 months), chicken (4 months), sheep (13 months), and goat (9 months), including the proximate composition, fatty acid composition, amino acids, and mineral contents. According to our findings, bovine and sheep bone extracts are more nutritious than goat and chicken bone extracts due to larger quantities of myristic acid; pentadecanoic acid; palmitic acid; isomargaric, oleic, eicosanoic, and stearic acids; important amino acids; and minerals. The in vivo results indicated that the different bone extracts are beneficial against T2DM. Whereas the bovine extract was the most active, the sheep, goat, and ultimately chicken extract were the least active. This study could result in the creation of a simple, noninvasive, low-cost, and reliable method, paving the way for potential early therapeutic applications in T2DM control. More research is needed to determine the bioavailability of these nutrients.

## Figures and Tables

**Table 1:** Solution preparation used for amino acid analysis present in bovine (12 months), chicken (4 months), sheep (13 months), and goat (9 months) femur bone extracts.

	Buffer A	Buffer B	Column Regeneration Solution	Sample Dilution Buffer
pH value	3.45	10.85		2.20
Normality	0.12	0.20	0.50	0.12
Tri-sodium citrate dihydrate	11.8 g	19.6 g		11.8 g
NaOH		3.1 g	20.0 g	
Citric acid	6.0 g			6.0 g
Boric acid		5.0 g		
Methanol	65 mL			
Thiodiglycol				14 mL
Hydrochloric acid 32%	6.5 mL			12 mL
EDTA			0.2 g	
Phenol	0.5 g			2.0 g
Final volume	1.0 L	1.0 L	1.0 L	1.0 L

**Table 2:** Parameters used in mineral analysis that are present in bovine (12 months), chicken (4 months), sheep (13 months), and goat (9 months) femur bone extracts.

Parameter	Settings
RF power	1.20 kW
Sampling depth	3.0 mm
Plasma gas	Ar 8.0 L/min
Auxiliary gas	Ar 1.10 L/min
Carrier gas	Ar 0.70 L/min
Torch	Minitorch
Nebulizer	Nebulizer, 07UES
Chamber	Cyclone chamber
Chamber temp.	5 °C
Cell gas (He)	6.0 mL/min
Cell voltage	−21.0 V
Energy filter	7 V

RF: radiofrequency, kW: Kilowatt, He: helium, V: voltage, mm: millimeter, L/min: liter per minute, mL/min: milliliter/minute, °C: degree Celsius.

**Table 3:** Proximate compositions (%) of bovine (12 months), chicken (4 months), sheep (13 months), and goat (9 months) femur bone extracts.

	Amount (mg/100 mg)			
	Bovine	Chicken	Sheep	Goat
Moisture	30,600 ± 0.001 ^d^	36,500 ± 0.008 ^b^	34,500 ± 0.003 ^c^	37,500 ± 0.009 ^a^
Crude protein	17,000 ± 0.008 ^a^	11,000 ± 0.002 ^c^	16,000 ± 0.003 ^b^	10,000 ± 0.001^d^
Crude fat	0.170 ± 0.006 ^b^	0.120 ± 0.008 ^c^	0.160 ± 0.005 ^a^	0.100 ± 0.004 ^d^

Data are expressed as mean ± SD (n = 3). One-way analysis of variance (ANOVA) was used in this statistical study, and the means of the therapy group were compared by using a post hoc and costate computer program. Using the SPSS (SPSS for Windows 7, version 8, Chicago, IL, USA) program, groups with similar letters inside the same row are not substantially different, whereas those with distinct letters are significantly different at *p* ≤ 0.05.

**Table 4:** Fatty acids composition of bovine (12 months), chicken (4 months), sheep (13 months), and goat (9 months) bone extracts using GC-MS analysis.

No.	Identified Compound	C:D	Type	Area %				RT	RI
				Bovine	Chicken	Sheep	Goat		
1	13,16-Octadecadiynoic acid	C18:2 (13, 16)	PUFA	0.80				4.04	707
2	Caprylic acid	C8:0	SFA		0.15		0.21	5.82	905
3	9-Oxononanoic acid	C9:0	SFA		0.52			14.84	824
4	Lauric acid	C12:0	SFA	0.41				16.99	846
5	Tridecanoic acid	C13:0	SFA	0.83				21.51	807
6	Myristic acid	C14:0	SFA		0.86	5.85	7.89	22.51	896
7	Pentadecanoic acid	C15:0	SFA	15.94		0.37		22.73	942
8	Succinic acid	C15:0	SFA	2.25		0.07		24.39	941
9	Palmitic acid	C16:0	SFA	19.30	33.89	23.62	12.07	26.66	969
10	Palmitoleic acid	C16:1 (9)	MUFA	4.26	0.82	0.35		27.10	911
11	Isomargaric acid	C17:0	SFA	7.48				29.43	832
12	Margaric acid	C17:0	SFA			2.92		30.23	851
13	Oleic acid	C18:1 (9)	MUFA	22.99	54.70 *	53.80 *	60.81 *	32.13	970
14	Eicosanoic acid	C20:0	SFA	25.70 *	1.01	0.84		32.74	937
15	Stearic acid	C18:0	SFA		5.04	8.64	9.03	34.10	924
16	6,9-Linoleic acid	C18:2 (6, 9)	PUFA		0.83		0.40	34.58	841
17	13,16-Octadecadiynoic acid	C18:2 (13, 16)	PUFA		0.04			34.94	814
18	6,9,11-Octadecatrienoic acid	C18:3 (6, 9, 11)	PUFA		1.00			35.26	851
19	8,11,14-Heptadecatrienoic acid	C17:3 (8, 11, 14)	PUFA		0.14			35.71	831
20	9,12,15-Octadecatrienoic acid	C18:3 (9, 12, 15)	PUFA		0.33		0.33	35.97	845
21	11-Eicosenoic acid	C20:1 (11)	MUFA			0.35	1.28	36.17	884
22	Cholestan-3-ol, 2-methylene-	C28:1	Sterol		0.16		0.89	37.20	778
23	6-Hexadecenoic acid, 7-methyl	C17:1 (6)	MUFA				1.39	37.61	764
24	8,11,14-Eicosatrienoic acid	C20:3 (8, 11, 14)	PUFA			0.11		37.72	804
25	5,8,11,14-Eicosatetraenoic acid	C20:4 (5, 8, 11, 14)	PUFA		0.14	0.12		37.76	885
26	9,12-Linoleic acid	C18:2 (9, 12)	PUFA		0.10	1.73		38.71	889
27	Cholest-5-en-3-yl myristate	C41:1	Sterol			0.11		46.12	823
28	Cholest-5-en-3-ol (3*α*), propanoate	C30:1	Sterol		0.10			46.14	775
29	Cholesta-3,5-diene	C27:2	Sterol				0.37	46.36	886
SFA				71.91%	41.47%	42.31%	29.2%		
USFA				28.05%	58.10%	56.46%	64.21%		
MUFA				27.25%	55.52%	54.50%	63.48%		
PUFA				0.80%	2.58%	1.96%	0.73%		
Sterol					0.26%	0.11%	1.26%		
Total				99.96%	99.83%	98.88%	94.67%		

RT: retention time for fatty acid in ester form (min), RI: retention index relative to *n*-alkanes, C:D: carbon number to double bond number involving their position, SFA: saturated fatty acid, USFA: unsaturated fatty acid, MUFA: monounsaturated fatty acid, PUFA: polyunsaturated fatty acid, *: major compound, %: percentage.

**Table 5:** Amino acid content of bovine (12 months), chicken (4 months), sheep (13 months), and goat (9 months) bone extracts.

No.	Identified Compound	RT	Amount (mg/100 mg)			
			Bovine	Chicken	Sheep	Goat
1	Aspartic acid	7.699	0.080 ± 0.02 ^a^	0.254 ± 0.03 ^c^	0.093 ± 0.01 ^a^	0.122 ± 0.03 ^b^
2	Threonine	9.816	0.052 ± 0.01 ^a^	0.131 ± 0.03 ^b^	0.045 ± 0.03 ^a^	0.235 ± 0.03 ^c^
3	Serine	10.549	0.046 ± 0.01 ^a^	0.093 ± 0.01 ^a^	0.070 ± 0.03 ^a^	0.076 ± 0.03 ^a^
4	Glutamic acid	11.915	0.134 ± 0.03 ^a^	0.681 ± 0.03 ^b^	0.180 ± 0.01 ^a^	0.129 ± 0.02 ^a^
5	Proline	13.933	1.532 ± 0.01 ^b^	1.876 ± 0.04 ^b^	0.873 * ± 0.02 ^a^	3.570 ± 0.03 ^c^
6	Glycine	17.824	1.626 * ± 0.03 ^b^	2.688 * ± 0.03 ^c^	0.714 ± 0.03 ^a^	4.254 * ± 0.01 ^d^
7	Alanine	19.056	1.130 ± 0.01 ^b^	1.746 ± 0.03 ^b^	0.458 ± 0.03 ^a^	3.370 ± 0.03 ^c^
8	Cystine	21.264		0.761 ± 0.01 ^a^		
9	Valine	21.915	0.327 ± 0.01 ^b^	0.417 ± 0.03 ^c^	0.189 ± 0.01 ^a^	0.701 ± 0.03 ^d^
10	Methionine	23.803	0.283 ± 0.03 ^a^	0.354 ± 0.05 ^b^	0.281 ± 0.01 ^a^	0.295 ± 0.03 ^a^
11	Isoleucine	25.915	0.158 ± 0.02 ^a^	0.357 ± 0.03 ^c^	0.280 ± 0.01 ^b^	0.366 ± 0.02 ^c^
12	Leucine	27.171	0.465 ± 0.03 ^b^	0.516 ± 0.03 ^c^	0.199 ± 0.03 ^a^	1.284 ± 0.01 ^d^
13	Tyrosine	30.315	0.076 ± 0.03 ^a^	0.067 ± 0.03 ^a^	0.043 ± 0.02 ^a^	0.202 ± 0.01 ^b^
14	Phenylalanine	31.389	0.241 ± 0.03 ^b^	0.379 ± 0.01 ^c^	0.083 ± 0.03 ^a^	0.623 ± 0.03 ^d^
15	Histidine	35.128	0.233 ± 0.01 ^c^	0.136 ± 0.03 ^b^	0.087 ± 0.03 ^a^	0.522 ± 0.04 ^d^
16	Lysine	39.381	0.258 ± 0.03 ^b^	0.377 ± 0.03 ^c^	0.060 ± 0.03 ^a^	0.978 ± 0.01 ^d^
17	Arginine	43.056	0.598 ± 0.02 ^b^	0.920 ± 0.01 ^c^	0.168 ± 0.01 ^a^	1.983 ± 0.05 ^d^
Total AA			7.237	11.753	3.823	18.708

Data are expressed as mean ± SD (n = 3). To compare the therapy group means, one-way analysis of variance (ANOVA) is used, followed by a post hoc and costate computer program. Using the SPSS (SPSS for Windows 7, version 8, Chicago, IL, USA) program, groups with similar letters inside the same row are not substantially different, whereas those with distinct letters are significantly different at *p* ≤ 0.05. RT: retention time for amino acid (min), AA: amino acid, *: major compound, %: percentage.

**Table 6:** Mineral contents of bovine (12 months), chicken (4 months), sheep (13 months), and goat (9 months) femur extracts.

No.	Identified Element	Amount (mg/100 mg)				RDI	MDI	PMTDI
		Bovine	Chicken	Sheep	Goat			
1	Al	0.105 ± 0.02 ^a^	0.077 ± 0.01 ^b^	0.084 ± 0.02 ^b^	0.116 ± 0.02 ^a^			4.3 mg/kg/d
2	Cd	0.051 ± 0.04 ^a^	0.038 ± 0.03 ^a^	0.046 ± 0.01 ^a^	0.067 ± 0.02 ^a^			1 mg/kg/d
3	Pb	0.982 ± 0.02 ^b^	0.776 ± 0.07 ^c^	0.912 ± 0.04 ^b^	1.054 ± 0.02 ^a^			3.57 mg/kg/d
4	Cr	6.010 ± 0.02 ^a^	5.600 ± 0.01 ^b^	5.600 ± 0.02 ^b^	6.700 ± 0.03 ^a^	25 µg (W), 35 µg (M)	200 µg	100 mg/kg/d
5	Ca	2.722 ± 0.01 ^b^	2.181 ± 0.02 ^b^	2.475 ± 0.05 ^b^	3.000 ± 0.02 ^a^	700 mg	2.500 mg	
6	Co	0.195 ± 0.02 ^b^	0.093 ± 0.02 ^c^	0.126 ± 0.02 ^b^	0.288 ± 0.02 ^a^	5–8 µg	8 µg/kg b.wt./d	
7	Cu	0.016 ± 0.02 ^a^	0.082 ± 0.02 ^a^	0.012 ± 0.02 ^a^	0.019 ± 0.02 ^a^	900 µg	1.400–1.700 µg	
8	Fe	0.750 ± 0.01 ^b^	0.701 ± 0.04 ^b^	0.634 ± 0.02 ^c^	0.850 ± 0.07 ^a^	14.8 mg (W), 8.7 mg (M)	45 mg	
9	Mg	2.530 ± 0.01 ^a^	2.250 ± 0.02 ^a^	2.100 ± 0.02 ^a^	2.712 ± 0.02 ^a^	310–320 mg (W), 400–420 mg (M)	>420 mg	
10	P	1.156 ± 0.03 ^a^	0.915 ± 0.06 ^b^	1.006 ± 0.02 ^a^	1.370 ± 0.02 ^a^	1.189 mg (W), 1.596 mg (M)	4.000 mg	
11	Zn	0.038 ± 0.02 ^a^	0.020 ± 0.05 ^a^	0.031 ± 0.02 ^a^	0.043 ± 0.09 ^a^	8 mg (W), 11 mg (M)	40 mg	

Data are expressed as mean ± SD (n = 3). Statistical analysis is carried out by using one-way analysis of variance (ANOVA) followed by a post hoc and costate computer program for comparison of the means of the therapeutic group. Groups with similar letters within same row are not significantly different, while those with different letters are significantly different at *p* ≤ 0.05, found by using SPSS (SPSS for Windows 7, version 8, Chicago, IL, USA) software. RDI: recommended daily intake for adults up to 50 years old; MDI: maximum daily intake for adults up to 50 years old; PMTDI: provisional maximum tolerable daily intake, which is the endpoint used for contaminants with no cumulative properties. Its value represents permissible human exposure because of the natural occurrence of the substance in food and in drinking water; W: women, M: men, Al: aluminum, Cd: cadmium, Pb: lead, Cr: chromium, Ca: calcium, Co: cobalt, Cu: copper, Fe: iron, Mg: magnesium, P: phosphorus, Zn: zinc.

**Table 7:** DPPH scavenging activity of bovine (12 months), chicken (4 months), sheep (13 months), and goat (9 months) bone extracts.

Extract	Concentration	
	0.01 µg/mL	0.05 µg/mL
Bovine	31.80 ± 1.11 ^a^	88.6 ± 7.00 ^b^
Chicken	6.80 ± 0.13 ^a^	16.00 ± 0.22 ^b^
Sheep	18.00 ± 0.44 ^a^	25.00 ± 1.90 ^b^
Goat	10.00 ± 0.03 ^a^	18.00 ± 0.04 ^b^
Vitamin C	82.00 ± 3.00 ^a^	88.00 ± 4.90 ^a^

Data are expressed as mean ± SD (n = 3). To compare the means of the therapy group, one-way analysis of variance (ANOVA) is used, followed by post hoc and costate computer programs. Using the SPSS (SPSS for Windows 7, version 8, Chicago, IL, USA) program, groups with similar letters are not significantly different, whereas those with distinct letters are significantly different at *p* ≤ 0.05.

**Table 8:** Blood sugar levels in different therapeutic groups: bovine (12 months), chicken (4 months), sheep (13 months), and goat (9 months) femur bone extracts.

Extracts and Standard Drug	Control Rats	Diabetic Rats	Treated Rats
% Change	110 ± 5.30 ^a^	350 ± 12.00 ^b^ 218.18	
Bovine % Improvement	-	-	155 ± 6.00 ^c^ 177.30
Chicken % Improvement	-	-	179 ± 10.12 ^d^ 155.50
Sheep % Improvement	-	-	160.00 ± 8.30 ^c^ 172.73
Goat % Improvement	-	-	175 ± 5.74 ^d^ 159.10
Glibenclamide % Improvement	-	-	140 ± 3.00 ^e^ 190.91

Data are expressed as mean ± SD (n = 10). For the purpose of comparing the means of the therapy group, one-way analysis of variance (ANOVA) is used, followed by post hoc and costate computer programs. Using the SPSS (SPSS for Windows 7, version 8, Chicago, IL, USA) program, groups with similar letters are not significantly different, whereas those with distinct letters are significantly different at *p* ≤ 0.05. $\%\;change=\frac{mean\;of\;negative\;control-mean\;of\;treatment\;group}{mean\;of\;negative\;control}\times 100$, $\%\;improvement=\frac{mean\;of\;positive\;control-mean\;of\;treatment\;group}{mean\;of\;negative\;control}\times 100$.

**Table 9:** Effect of different extracts (bovine (12 months), chicken (4 months), sheep (13 months), and goat (9 months) femur extracts) orally administered on lipid profile (mg/dL) in rats with diabetes induced by streptozotocin (STZ).

Groups/Parameters	TL	TG	TC	HDL-C
Control	600.00 ± 19.00 ^a^	102.00 ± 4.00 ^c^	110.00 ± 8.00 ^a^	50.00 ± 2.00 ^a^
STZ-diabetic rats	1250.00 ± 30.00 ^b^	250.00 ± 10.00 ^b^	279.00 ± 9.20 ^b^	19.10 ± 1.00 ^b^
% Change	108.33	145.10	153.64	61.80
Bovine	660.00 ± 20.00 ^c^	180.00 ± 3.00 ^a^	153.00 ± 8.00 ^a^	31.00 ± 2.00 ^b^
% Improvement	98.33	68.63	114.55	24.00
Chicken	950.00 ± 21.00 ^a^	200.00 ± 8.00 ^c^	191.00 ± 5.00 ^a^	23.00 ± 2.77 ^c^
% Improvement	50.00	49.01	80.00	8.00
Sheep	680.00 ± 15.00 ^d^	185.00 ± 5.00 ^d^	169.00 ± 5.20 ^d^	28.00 ± 1.00 ^c^
% Improvement	95.00	63.73	100.00	17.80
Goat	900.00 ± 22.00 ^c^	199.00 ± 4.00 ^a^	180.00 ± 5.00 ^c^	25.00 ± 1.10 ^b^
% Improvement	58.33	50.00	90.00	12.00
Glibenclamide	650.00 ± 28.70 ^ac^	111.20 ± 6.00 ^ac^	150.00 ± 6.00 ^a^	37.00 ± 3.20 ^a^
% Improvement	100.00	136.07	117.27	35.80

Data are expressed as mean ± SD (n = 10). For the purpose of comparing the means of the therapy group, one-way analysis of variance (ANOVA) is used, followed by post hoc and costate computer programs. Using the SPSS (SPSS for Windows 7, version 8, Chicago, IL, USA) program, groups with similar letters are not significantly different, whereas those with distinct letters are significantly different at *p* ≤ 0.05. $\%\;change=\frac{mean\;of\;negative\;control-mean\;of\;treatment\;group}{mean\;of\;negative\;control}\times 100$, $\%\;improvement=\frac{mean\;of\;positive\;control-mean\;of\;treatment\;group}{mean\;of\;negative\;control}\times 100$. TL: total lipid, TG: triglycerides, TC: serum total cholesterol, HDL-C: high-density lipoprotein cholesterol.

**Table 10:** Effect of different extracts—bovine (12 months), chicken (4 months), sheep (13 months), and goat (9 months) femur bone extracts—orally administered on liver function in rats with diabetes induced by streptozotocin (STZ).

Groups/Parameters	ALT (U/L)	AST(U/L)	ALP (U/L)	Bilirubin (mg/dL)	Total Protein (μg/mL)
Control	85.00 ± 2.66	144.00 ± 8.20 ^a^	90.00 ± 5.00 ^a^	0.54 ± 0.05 ^a^	5.40 ± 0.43 ^a^
STZ-Diabetic Rats	230.00 ± 10.00 ^b^	265.00 ± 10.00 ^b^	190.00 ± 10.00 ^b^	1.40 ± 0.12 ^b^	2.00 ± 0.40 ^b^
% Change	170.56	84.03	111.11	159.30	62.96
Bovine	150.00 ± 8.10 ^c^	165.00 ± 6. 00 ^c^	140.00 ± 9.00 ^c^	0.90 ± 0.25 ^c^	3.98 ± 0.24 ^c^
% Improvement	94.12	69.44	55.56	92.59	36.67
Chicken	190.00 ± 9.66 ^a^	180.00 ± 9.00 ^a^	160.00 ± 8.12 ^a^	1.07 ± 0.30 ^a^	2.69 ± 0.41 ^a^
% Improvement	47.10	59.03	33.33	61.11	12.78
Sheep	182.55 ± 10.00 ^d^	169.00 ± 6.00 ^a^	144.00 ± 6.10 ^d^	1.00 ± 0.50 ^d^	2.34 ± 0.33 ^a^
% Improvement	55.83	66.67	51.11	74.10	6.30
Goat	189.00 ± 6.00 ^c^	178.00 ± 6.00 ^c^	151.00 ± 9.00 ^c^	1.02 ± 0.13 ^a^	2.89 ± 0.20 ^a^
% Improvement	48.34	60.42	43.33	70.37	16.48
Glibenclamide	89.00 ± 9.70 ^a^	159.00 ± 8.00 ^a^	133.00 ± 3.60 ^a^	0.59 ± 0.32 ^a^	4.90 ± 0.30 ^a^
% Improvement	165.88	73.61	63.33	150.00	53.70

Data are expressed as mean ± SD (n = 10). For the purpose of comparing the means of the therapy group, one-way analysis of variance (ANOVA) is used, followed by post hoc and costate computer programs. Using the SPSS (SPSS for Windows 7, version 8, Chicago, IL, USA) program, groups with similar letters are not significantly different, whereas those with distinct letters are significantly different at *p* ≤ 0.05. $\%\;change=\frac{mean\;of\;negative\;control-mean\;of\;treatment\;group}{mean\;of\;negative\;control}\times 100$, $\%\;improvement=\frac{mean\;of\;positive\;control-mean\;of\;treatment\;group}{mean\;of\;negative\;control}\times 100$. AST: serum aspartate aminotransferase, ALP: alkaline phosphatase, ALT: alanine aminotransferase.

**Table 11:** Effect of orally administered extracts—bovine (12 months), chicken (4 months), sheep (13 months), and goat (9 months) femur bone extracts—on kidney function (mg/dL) in rats with diabetes induced by streptozotocin (STZ).

Groups/Parameters	Creatinine	Urea
Control	0.34 ± 0.02 ^b^	34.00 ± 2.00 ^b^
STZ-diabetic rats	0.79 ± 0.04 ^a^	99.00 ± 4.00 ^a^
% Change	132.35	191.18
Bovine	0.50 ± 0.03 ^c^	50.00 ± 2.12 ^c^
% Improvement	85.29	144.12
Chicken	0.64 ± 0.04 ^b^	72.00 ± 3.22 ^b^
% Improvement	44.12	79.41
Sheep	0.56 ± 0.03 ^b^	52.00 ± 4.00 ^c^
% Improvement	67.65	138.24
Goat	0.60 ± 0.03 ^a^	69.00 ± 2.00 ^a^
% Improvement	55.88	88.23
Glibenclamide	0.39 ± 0.06 ^b^	43.00 ± 2.11 ^b^
% Improvement	117.65	164.71

Data are expressed as mean ± SD (n = 10). For the purpose of comparing the means of the therapy group, one-way analysis of variance (ANOVA) is used, followed by post hoc and costate computer programs. Using the SPSS (SPSS for Windows 7, version 8, Chicago, IL, USA) program, groups with similar letters are not significantly different, whereas those with distinct letters are significantly different at *p* ≤ 0.05. $\%\;change=\frac{mean\;of\;negative\;control-mean\;of\;treatment\;group}{mean\;of\;negative\;control}\times 100$, $\%\;improvement=\frac{mean\;of\;positive\;control-mean\;of\;treatment\;group}{mean\;of\;negative\;control}\times 100$.

**Table 12:** Effect of different extracts (bovine (12 months), chicken (4 months), sheep (13 months), and goat (9 months) femur bone extracts) orally administered on proinflammatory markers in rats with diabetes induced by streptozotocin (STZ).

Groups/Parameters	IL-6 (pg/mL)	TNF-α (pg/mL)	sICAM-1 (pg/mL)	sVCAM-1 (pg/mL)
Control	99.00 ± 5.00 ^a^	110.00 ± 9.00 ^a^	330.00 ± 11.00 ^a^	1200 ± 200.00 ^a^
STZ-diabetic rats	266.00 ± 11.00 ^b^	240.00 ± 10.00 ^b^	1200.00 ± 15.00 ^b^	21,900 ± 300.00 ^b^
% Change	168.69	118.18	63.64	1725.00
Bovine	190.00 ± 5.10 ^c^	194.00 ± 5.50 ^c^	600.00 ± 28.00 ^c^	10,500 ± 300.00 ^c^
% Improvement	76.77	41.82	181.82	950.00
Chicken	210.80 ± 5.10 ^a^	105.00 ± 5.34 ^a^	1000.00 ± 15.00 ^d^	12,000 ± 380.00 ^d^
% Improvement	55.76	122.73	60.61	825.00
Sheep	193.00 ± 6.00 ^d^	135.00 ± 6.30 ^d^	700.00 ± 10.00 ^d^	12,900 ± 230.00 ^d^
% Improvement	73.74	95.45	151.52	750.00
Goat	200.00 ± 8.00 ^c^	195.00 ± 5.98 ^c^	900.00 ± 22.00 ^b^	11,000 ± 350.00 ^b^
% Improvement	66.67	40.91	90.91	908.33
Glibenclamide	120.00 ± 9.00 ^d^	140.00 ± 10.00 ^d^	567.00 ± 18.90 ^d^	11,700 ± 160.00 ^a^
% Improvement	147.47	90.91	191.82	850.00

Data are expressed as mean ± SD (n = 10). For the purpose of comparing the means of the therapy group, one-way analysis of variance (ANOVA) is used, followed by post hoc and costate computer programs. Using the SPSS (SPSS for Windows 7, version 8, Chicago, IL, USA) program, groups with similar letters are not significantly different, whereas those with distinct letters are significantly different at *p* ≤ 0.05. $\%\;change=\frac{mean\;of\;negative\;control-mean\;of\;treatment\;group}{mean\;of\;negative\;control}\times 100$, $\%\;improvement=\frac{mean\;of\;positive\;control-mean\;of\;treatment\;group}{mean\;of\;negative\;control}\times 100$. IL-6: interleukin-6, TNF-α: tumor necrosis factor-alpha, sICAM-1: serum soluble intercellular adhesion molecule-1, and sVCAM-1: serum vascular cell adhesion molecule-1.

**Table 13:** Effect of different orally administered extracts—bovine (12 months), chicken (4 months), sheep (13 months), and goat (9 months) bone extracts orally administered—on oxidative stress assessment levels (MDA and GSH) in rats with diabetes induced by streptozotocin (STZ).

Groups/Parameters	MDA (nmol/g Tissue)	GSH (nmol/g Tissue)
Control	110.00 ± 5.00 ^a^	750.00 ± 12.00 ^a^
STZ-Diabetic Rats	450.00 ± 11.00 ^b^	200.00 ± 5.00 ^b^
% Change	309.10	73.33
Bovine	200.00 ± 15.10 ^c^	520.00 ± 10.00 ^c^
% Improvement	227.27	42.67
Chicken	310.00 ± 9.22 ^a^	313.00 ± 10.00 ^a^
% Improvement	127.27	15.10
Sheep	230.00 ± 6.00 ^d^	510.00 ± 6.00 ^d^
% Improvement	200.00	41.33
Goat	256.00 ± 7.00 ^c^	366.00 ± 10.00 ^c^
% Improvement	176.36	22.13
Glibenclamide	119.00 ± 6.10 ^a^	570.00 ± 10.00 ^d^
% Improvement	300.91	49.33

Data are expressed as mean ± SD (n = 10). For the purpose of comparing the means of the therapy group, one-way analysis of variance (ANOVA) is used, followed by post hoc and costate computer programs. Using the SPSS (SPSS for Windows 7, version 8, Chicago, IL, USA) program, groups with similar letters are not significantly different, whereas those with distinct letters are significantly different at *p* ≤ 0.05. $\%\;change=\frac{mean\;of\;negative\;control-mean\;of\;treatment\;group}{mean\;of\;negative\;control}\times 100$, $\%\;improvement=\frac{mean\;of\;positive\;control\;–\;mean\;of\;treatment\;group}{mean\;of\;negative\;control}\times 100$. MDA: malondialdehyde, GSH: glutathione.

**Table 14:** Scoring of histopathological alterations in liver and pancreas of all treated groups—bovine (12 months), chicken (4 months), sheep (13 months), and goat (9 months) bone extracts.

Lesion	Control Group	Positive Control	Bovine	Chicken	Sheep	Goat	Standard Drug
Liver							
Vacuolar degeneration of hepatocytes	0	3	1	2	1	2	1
Pancreas							
Atrophy of islets of Langerhans	0	3	1	2	1	2	1
Periductal fibrosis	0	2	1	1	1	1	1
Vacuolation of exocrine pancreas	0	2	0	1	1	1	1
Necrosis of exocrine pancreas	0	3	1	2	2	1	1

The score system was designed as follows: score 0 (absence of the lesion in all rats of the group), score 1 (<30%), score 2 (30–50%), and score 3 (>50%). G1: control; G2: diabetes; G3–G6: diabetic rats treated with bovine (12 months), chicken (4 months), sheep (13 months), and goat (9 months) femur bone extracts, respectively; G7: diabetic rats treated with standard drug.

## Data Availability

Not applicable.

## Supplementary Materials

The following supporting information can be downloaded at https://www.mdpi.com/article/10.3390/nu15184037/s1, Figure S1. GC/MS spectrum for bovine femur bone fatty acids extract; Figure S2. GC/MS spectrum for chicken femur bone fatty acids extract; Figure S3. GC/MS spectrum for sheep femur bone fatty acids extract; Figure S4. GC/MS spectrum for goat femur bone fatty acids extract; Figure S5. Amino acid spectrum for bovine femur bone extract; Figure S6. Amino acid spectrum for chicken femur bone extract; Figure S7. Amino acid spectrum for sheep femur bone extract; Figure S8. Amino acid spectrum for goat femur bone extract.

## List of Abbreviations

AA: amino acids, ADHD: autism and attention-deficit hyperactivity disorder, ALP: alkaline phosphatase, ALT: alanine aminotransferase, Al: aluminum, AST: serum aspartate aminotransferase, Ca: calcium, Cd: cadmium, Co: cobalt, Cr: chromium, Cu: copper, CVD: cardiovascular disease, DKD: diabetic kidney disease, DPP-4: dipeptidyl peptidase-4, DPPH: 2,2-diphenyl-1-picrylhydrazyl, ELISA: enzyme-linked immunoassay, FA: fatty acid, FAME: fatty acids methyl esters, Fe: iron, GAPS: gut and psychology syndrome, GLP-1: glucagon-like peptide-1, GSH: glutathione, GC/MS: gas chromatography–mass spectrometry, HDL-C: high-density lipoprotein cholesterol, ICP-OES: inductively coupled plasma optical emission spectrophotometry, IDF: International Diabetes Federation, IL-6: interleukin-6, IR: insulin resistance, MDA: malondialdehyde, MDI: maximum daily intake, Mg: magnesium, MUFA: monounsaturated fatty acids, P: phosphorus, Pb: lead, PMTDI: provisional maximum tolerable daily intake, PUFA: polyunsaturated fatty acid, RDI: recommended daily intake, ROS: reactive oxygen species, SFA: saturated fatty acids, SGLT2: sodium–glucose co-transporter 2, sICAM-1: serum soluble intercellular adhesion molecule-1, STZ: streptozotocin, sVCAM-1: serum vascular cell adhesion molecule-1, TC: serum total cholesterol, T2DM: Type 2 diabetes mellitus, TG: triglycerides, TL: total lipid, TNF-α: tumor necrosis factor-alpha, USFA: unsaturated fatty acids, Zn: zinc, β: beta.
