# Supplementary material for: Nutritional Composition and Anti-Type 2 Diabetes Mellitus Potential of Femur Bone Extracts from Bovine, Chicken, Sheep, and Goat: Phytochemical and In Vivo Studies
Source: Nutrients. 2023 Sep 18;15(18):4037. doi: 10.3390/nu15184037 (PMC10534695; doi:10.3390/nu15184037)
Supplement: Supplementary file 1 [file nutrients-15-04037-s001.zip › nutrients-2594189-supplementary.pdf]

SUPPLEMENTARY DATA

Nutritional Composition and Anti-Type 2 Diabetes Mellitus Potential of Femur Bone Extracts from Bovine, Chicken, Sheep, and Goat: Phytochemical and In Vivo Studies

Naseh A. Algehainy [1], Esraa M. Mohamed [2], Hanan F. Aly [3], Eman A. Younis [3], Faisal H. Altemani [1], Mohammad A. Alanazi [1], Gerhard Bringmann [4,*], Usama Ramadan Abdelmohsen [5,*] and Abeer H. Elmaidomy [6]

[1] Department of Medical Laboratory Technology, Faculty of Applied Medical Sciences, University of Tabuk, Tabuk 71491, Saudi Arabia; nalgehainy@ut.edu.sa (N.A.A.); faltemani@ut.edu.sa (F.H.A.); m.alenezi@ut.edu.sa (M.A.A.)
[2] Department of Pharmacognosy, Faculty of Pharmacy, Misr University for Science & Technology (MUST), Giza 12566, Egypt; esraakadrymohamed@gmail.com
[3] Department of Therapeutic Chemistry, National Research Centre (NRC), El-Bouth St., Cairo 12622, Egypt; hanan_abduallah@yahoo.com (H.F.A.); youniseman530@yahoo.com (E.A.Y.)
[4] Institute of Organic Chemistry, University of Würzburg, Am Hubland, 97074 Würzburg, Germany
[5] Department of Pharmacognosy, Faculty of Pharmacy, Deraya University, 7 Universities Zone, New Minia 61111, Egypt
[6] Department of Pharmacognosy, Faculty of Pharmacy, Beni-Suef University, Beni-Suef 62511, Egypt; abeer011150@pharm.bsu.edu.eg
* Correspondence: bringman@chemie.uni-wuerzburg.de (G.B.); usama.ramadan@mu.edu.eg (U.R.A.)

List of Contents

Figure S1. GC/MS spectrum for bovine femur bone fatty acids extract.

Figure S2. GC/MS spectrum for chicken femur bone fatty acids extract.

Figure S3. GC/MS spectrum for sheep femur bone fatty acids extract.

Figure S4. GC/MS spectrum for goat femur bone fatty acids extract.

Figure S5. Amino acid spectrum for bovine femur bone extract.

Figure S6. Amino acid spectrum for chicken femur bone extract.

Figure S7. Amino acid spectrum for sheep femur bone extract.

Figure S8. Amino acid spectrum for goat femur bone extract.

My GC-MS Report

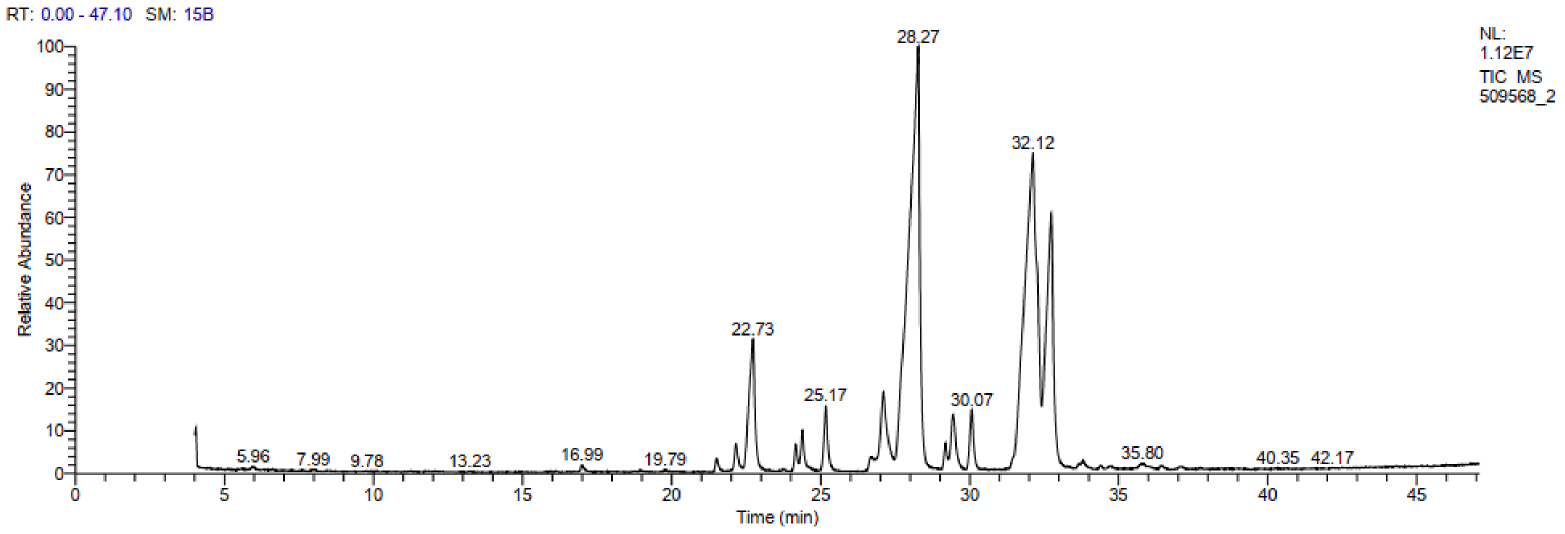

Figure S1. GC/MS spectrum for bovine femur bone fatty acids extract.

My GC-MS Report

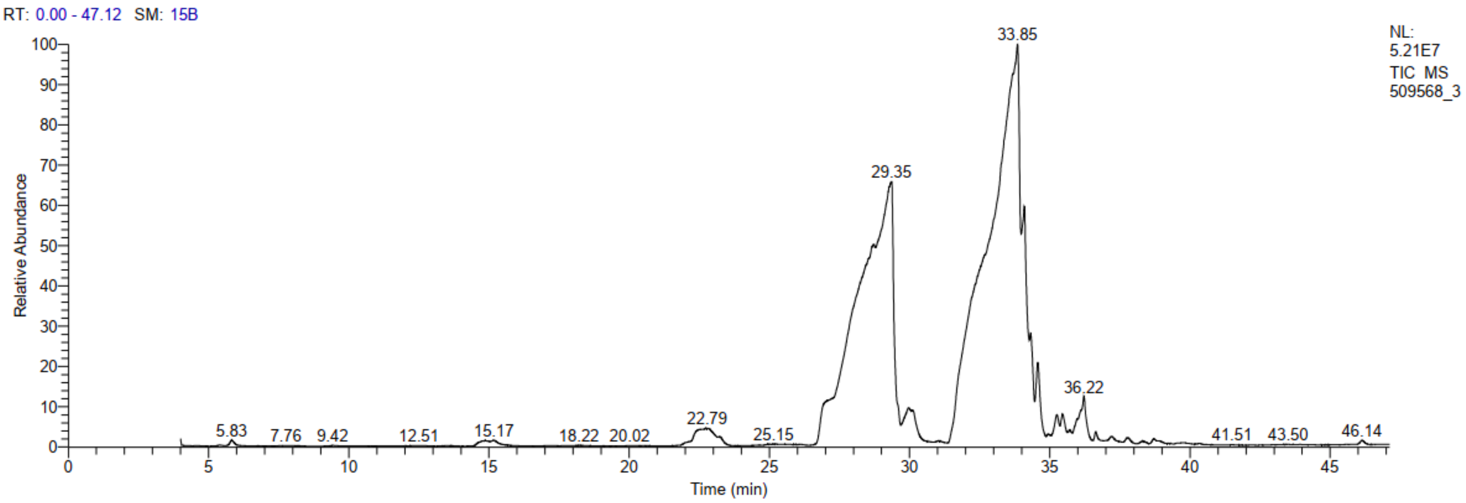

Figure S2. GC/MS spectrum for chicken femur bone fatty acids extract.

My GC-MS Report

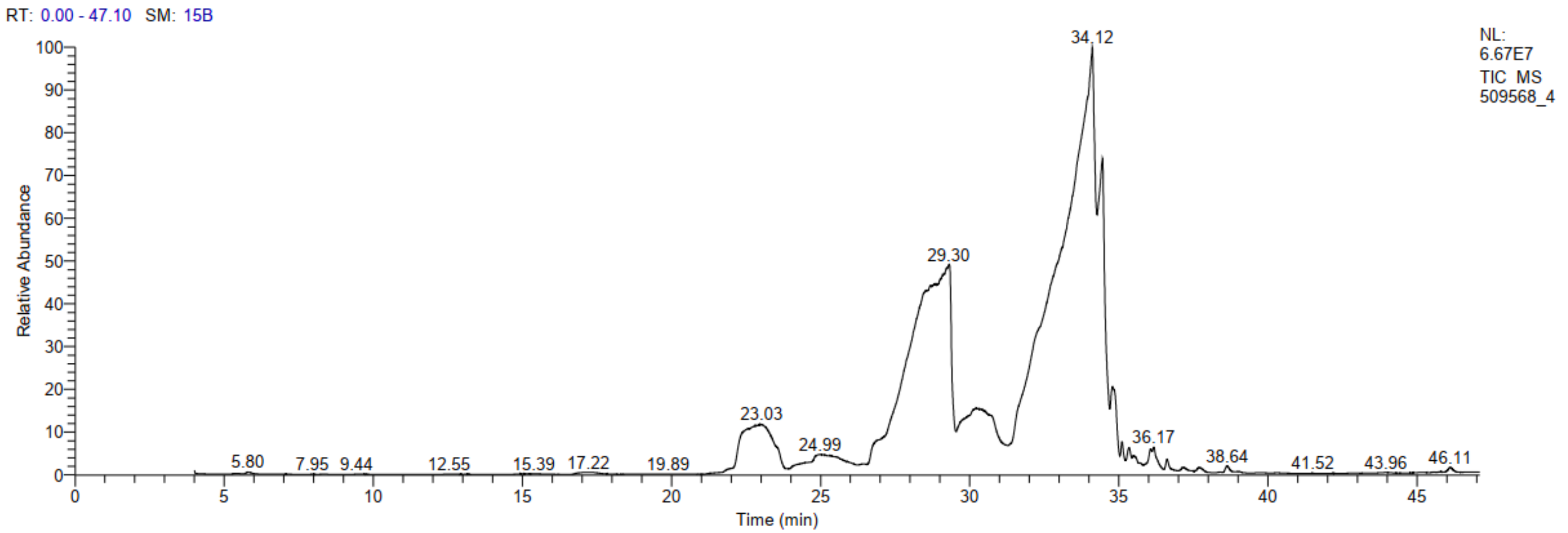

Figure S3. GC/MS spectrum for sheep femur bone fatty acids extract.

My GC-MS Report

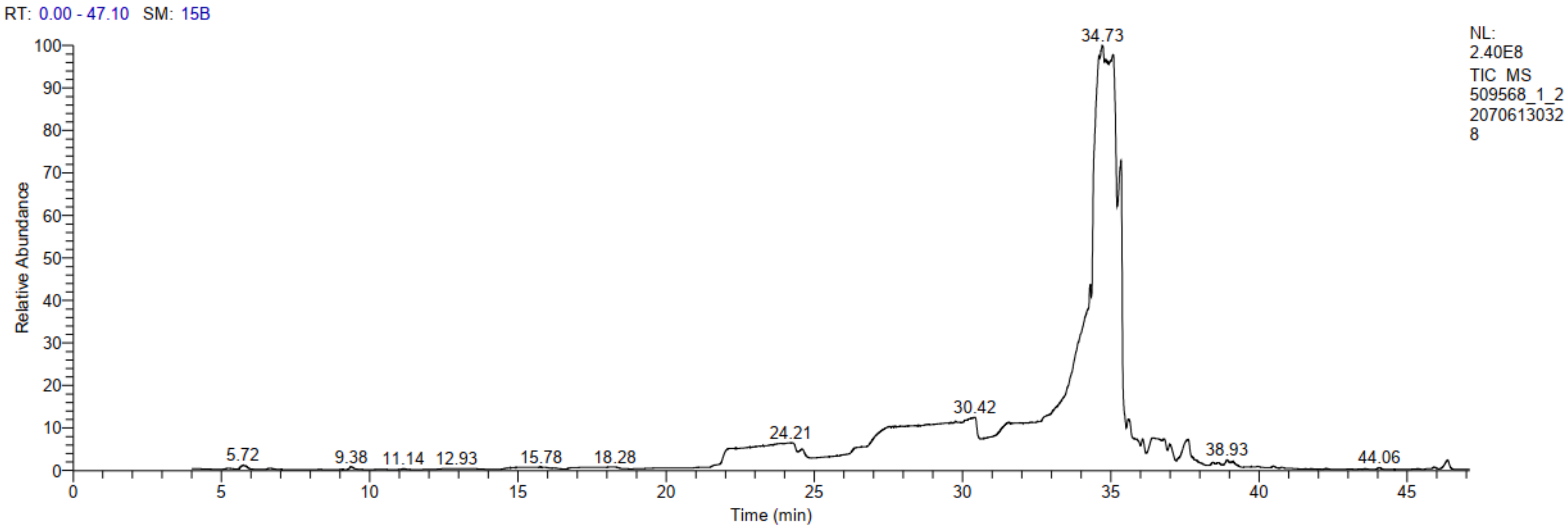

Figure S4. GC/MS spectrum for goat femur bone fatty acids extract.

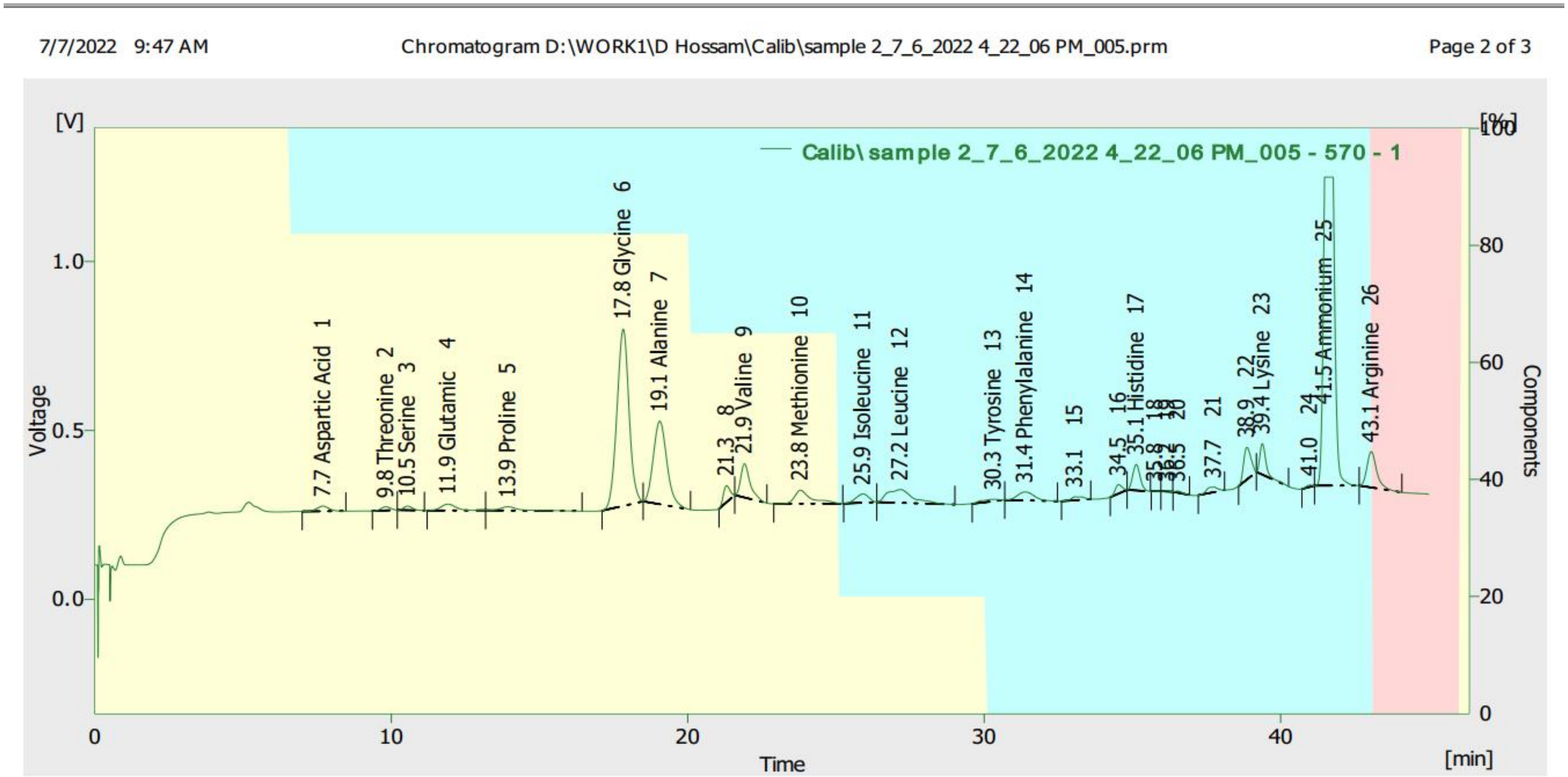

Figure S5. Amino acid spectrum for bovine femur bone extract.

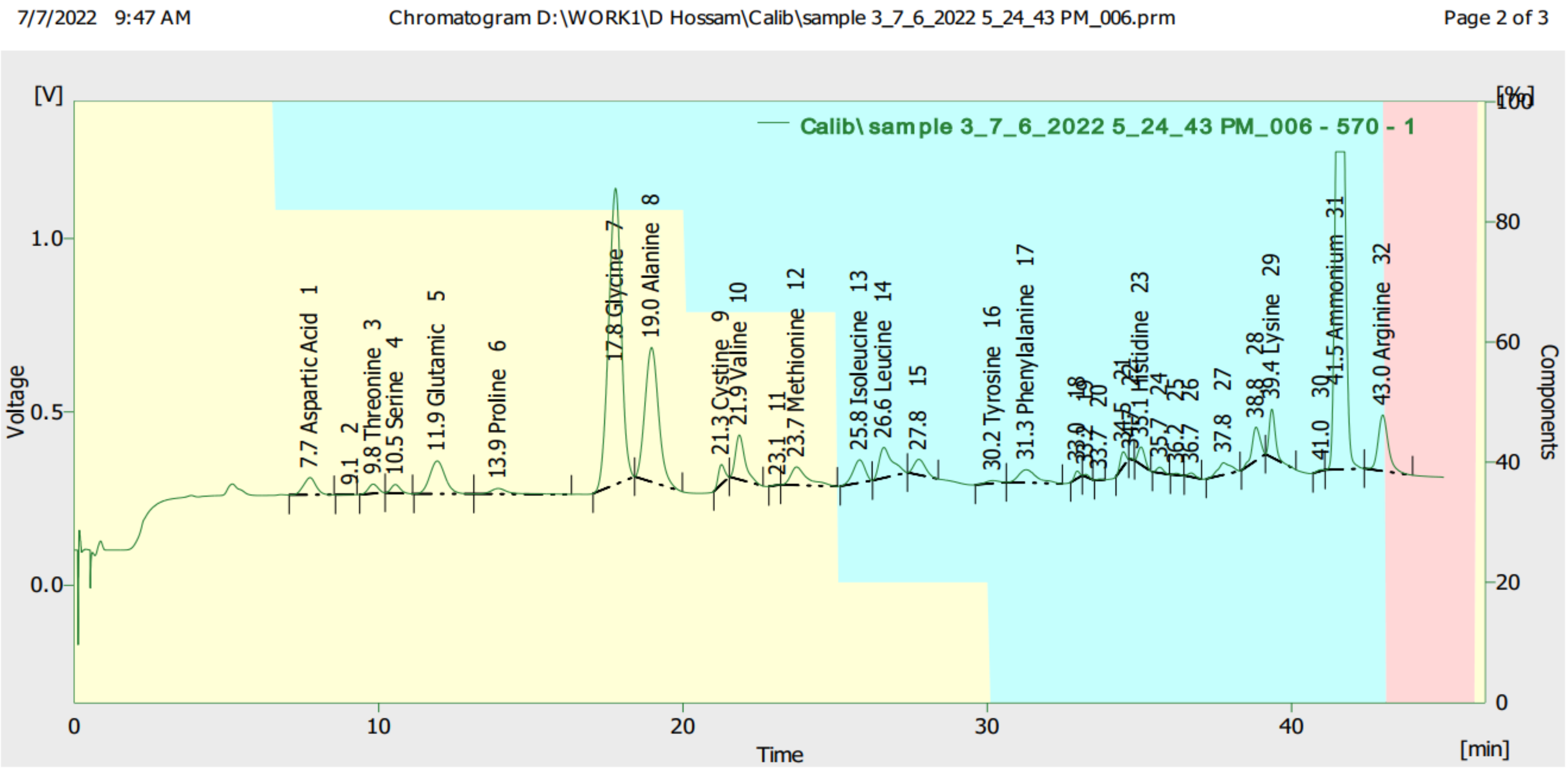

Figure S6. Amino acid spectrum for chicken femur bone extract.

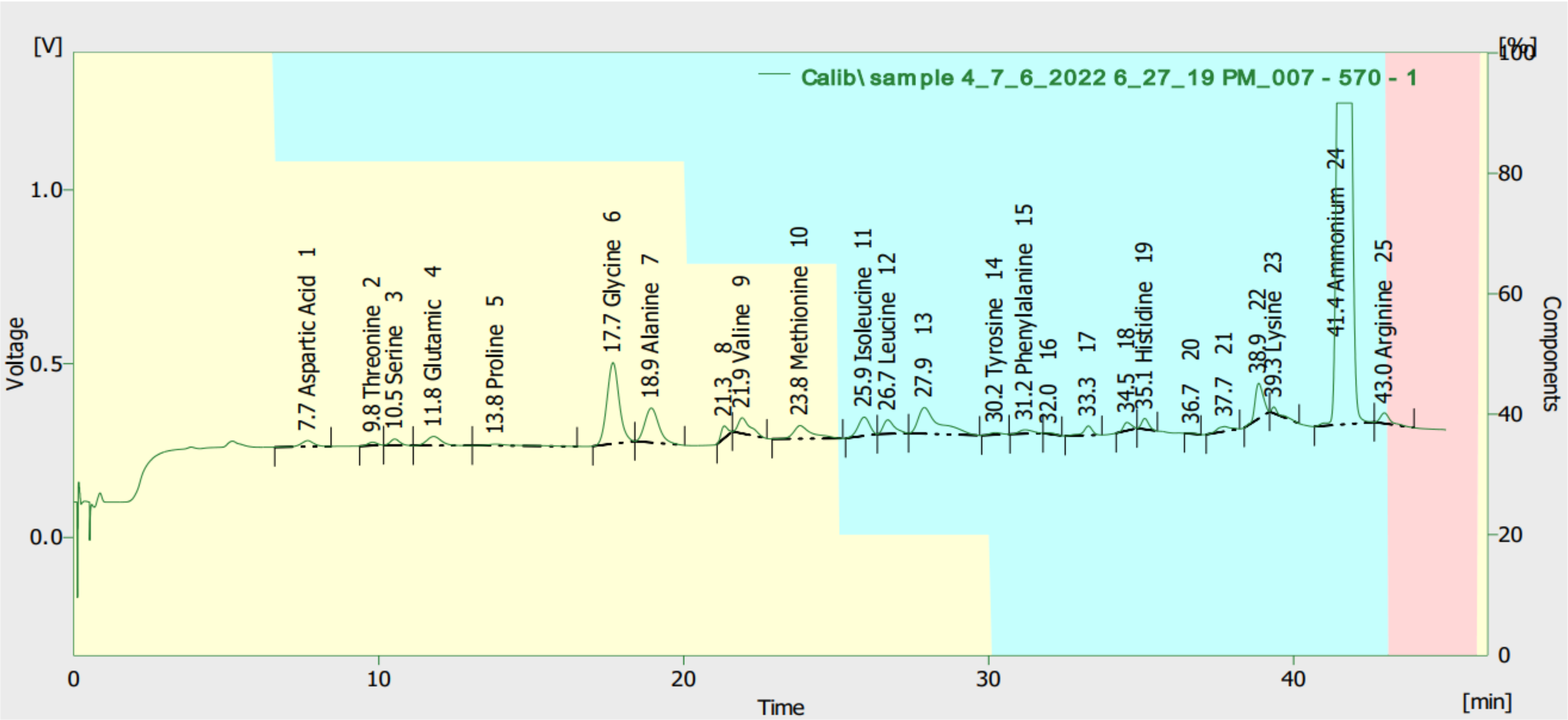

Figure S7. Amino acid spectrum for sheep femur bone extract.

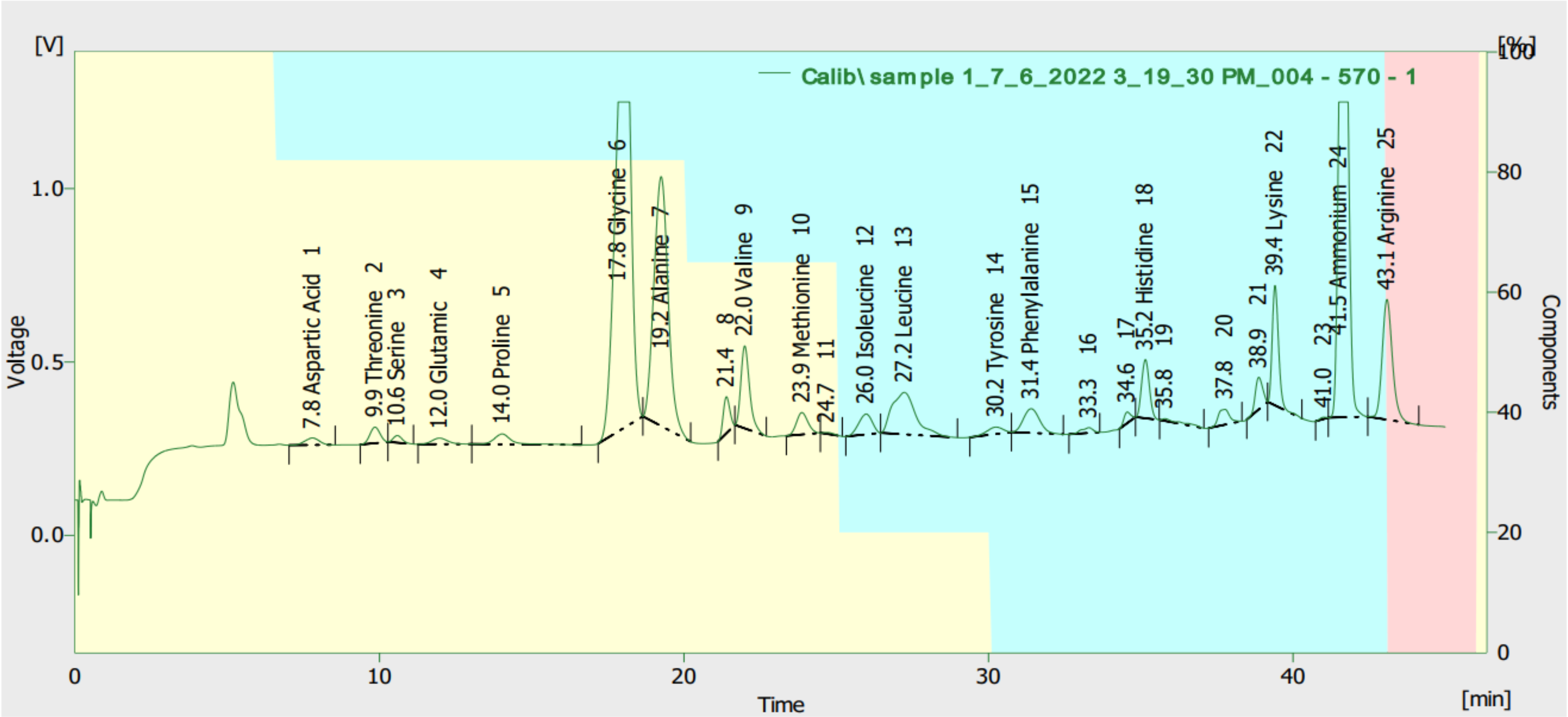

Figure S8. Amino acid spectrum for goat femur bone extract.
